# Epithelial miR-141 regulates IL-13–induced airway mucus production

**DOI:** 10.1172/jci.insight.139019

**Published:** 2021-03-08

**Authors:** Sana Siddiqui, Kristina Johansson, Alex Joo, Luke R. Bonser, Kyung Duk Koh, Olivier Le Tonqueze, Samaneh Bolourchi, Rodriel A. Bautista, Lorna Zlock, Theodore L. Roth, Alexander Marson, Nirav R. Bhakta, K. Mark Ansel, Walter E. Finkbeiner, David J. Erle, Prescott G. Woodruff

**Affiliations:** 1 Department of Medicine, Division of Pulmonary and Critical Care Medicine,; 2 Sandler Asthma Basic Research Center,; 3 Department of Microbiology and Immunology,; 4 Lung Biology Center,; 5 Cardiovascular Research Institute,; 6 Department of Pathology,; 7 Biomedical Sciences Graduate Program, and; 8 Diabetes Center, UCSF, San Francisco, California, USA.; 9 Innovative Genomics Institute, University of California, Berkeley, California, USA.; 10 J. David Gladstone Institutes, San Francisco, California, USA.; 11 Department of Medicine, Division of Infectious Diseases, UCSF, San Francisco, California, USA.; 12 Parker Institute for Cancer Immunotherapy, San Francisco, California, USA.; 13 Chan Zuckerberg Biohub, San Francisco, California, USA.

**Keywords:** Pulmonology, Asthma, Noncoding RNAs, Th2 response

## Abstract

IL-13–induced goblet cell metaplasia contributes to airway remodeling and pathological mucus hypersecretion in asthma. miRNAs are potent modulators of cellular responses, but their role in mucus regulation is largely unexplored. We hypothesized that airway epithelial miRNAs play roles in IL-13–induced mucus regulation. miR-141 is highly expressed in human and mouse airway epithelium, is altered in bronchial brushings from asthmatic subjects at baseline, and is induced shortly after airway allergen exposure. We established a CRISPR/Cas9-based protocol to target miR-141 in primary human bronchial epithelial cells that were differentiated at air-liquid-interface, and goblet cell hyperplasia was induced by IL-13 stimulation. miR-141 disruption resulted in decreased goblet cell frequency, intracellular MUC5AC, and total secreted mucus. These effects correlated with a reduction in a goblet cell gene expression signature and enrichment of a basal cell gene expression signature defined by single cell RNA sequencing. Furthermore, intranasal administration of a sequence-specific mmu-miR-141-3p inhibitor in mice decreased *Aspergillus*-induced secreted mucus and mucus-producing cells in the lung and reduced airway hyperresponsiveness without affecting cellular inflammation. In conclusion, we have identified a miRNA that regulates pathological airway mucus production and is amenable to therapeutic manipulation through an inhaled route.

## Graphical abstract

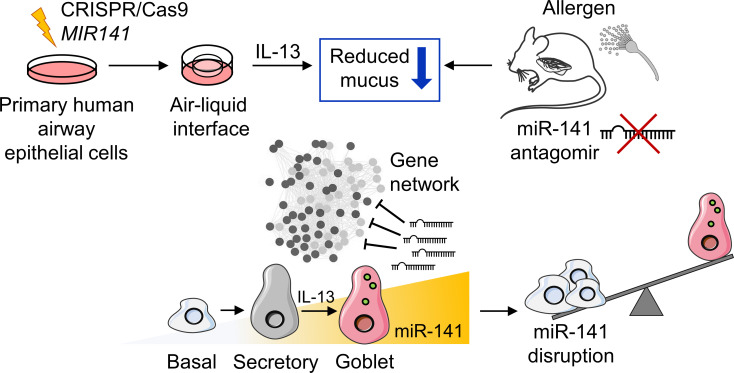

## Introduction

Epithelial cells form a barrier to the external environment and secrete mucus that traps inhaled particles and pathogens (1, 2). Defective epithelial function is a defining feature of asthma, and increased production of pathological mucus by airway epithelial cells can lead to mucus plugs that limit airflow (3, 4) and accumulate in asthma exacerbations (5). Airway goblet cells develop from basal cells and are specialized to produce, store, and release mucins and thereby play major roles in airway plugging. Despite the importance of mucus production in the pathophysiology of asthma and other respiratory diseases, there are currently no effective therapies that specifically target mucus overproduction in the airway.

Asthma is defined by chronic inflammation in the airway, which causes bronchial hyperreactivity and airflow obstruction (6, 7). Many people with asthma display evidence of a type 2–high (T2-high) phenotype with atopy and ongoing T2 airway inflammation (7) mediated by the cytokines IL-4, IL-5, and IL-13. While IL-4 and IL-5 drive IgE production and eosinophilia, respectively, IL-13 has important effects on structural cells including airway epithelial cells. IL-13 signaling through signal transducer and activator of transcription 6 (STAT6), subsequent engagement of the transcription factor SAM pointed domain–containing Ets transcription factor (SPDEF) (8), and alterations in the balance of forkhead box A2/A3 (FOXA2/FOXA3) (9) are critical steps in a major pathway for airway epithelial goblet cell metaplasia (10, 11). This pathway preferentially induces the mucin glycoprotein MUC5AC in vitro (12), recapitulating its preferential induction of MUC5AC in airway epithelial brushings from humans with T2-high asthma (7). MUC5AC may be particularly pathological given that it is poorly transported by the mucociliary apparatus and is the predominant mucin glycoprotein in fatal asthmatic airway plugs (2, 12).

miRNAs represent a distinct class of noncoding RNAs, approximately 20–22 nucleotides long, that mediate sequence-specific repression of target mRNAs, inhibiting gene expression at the posttranscriptional level (13). The seed sequence, situated at positions 2–7 from the miRNA 5′-end, mediates target recognition at sites typically within the 3′UTR of mRNAs (14). The most powerful and defining feature of miRNAs is their ability to regulate multiple target genes with related cellular functions. Thus, a single miRNA can have a major biological impact by acting as a master regulator of several genes in an inflammatory pathway (15). We have previously reported modulation of miRNAs, including miR-141, in bronchial epithelial brushings in asthmatics compared with healthy controls using miRNA arrays (16). Some miRNAs have been studied in relation to differentiation of ciliated cells, but published studies focusing on miRNAs related to mucus production are limited (17–19).

miR-141 belongs to the miR-141/200 family. miR-141 has not yet been reported to have a direct role in mucus production in asthma; however, it has a number of predicted mucus-related targets, including FOXA2 (18). LacZ reporter expression for murine miR-141 revealed a remarkable expression pattern that is observed almost exclusively in the adult murine airway, including the nasal cavity, trachea, bronchi, and bronchioles, although it is also present in the olfactory bulbs of the brain (20). This observation highlights that miR-141 is abundantly, and specifically, expressed in the branching airway and suggests that modulation of miR-141 may have an impact on cellular responses in airway diseases such as asthma. Here, we investigate a role for miR-141 in airway epithelial mucus production in primary human bronchial epithelial cell (HBEC) cultures using CRISPR/Cas9 gene editing and in an experimental mouse model of allergen-induced asthma using in vivo antagomir administration.

## Results

### miR-141 is highly expressed in human airway epithelial cells and dysregulated in asthma.

**Figure 1:**
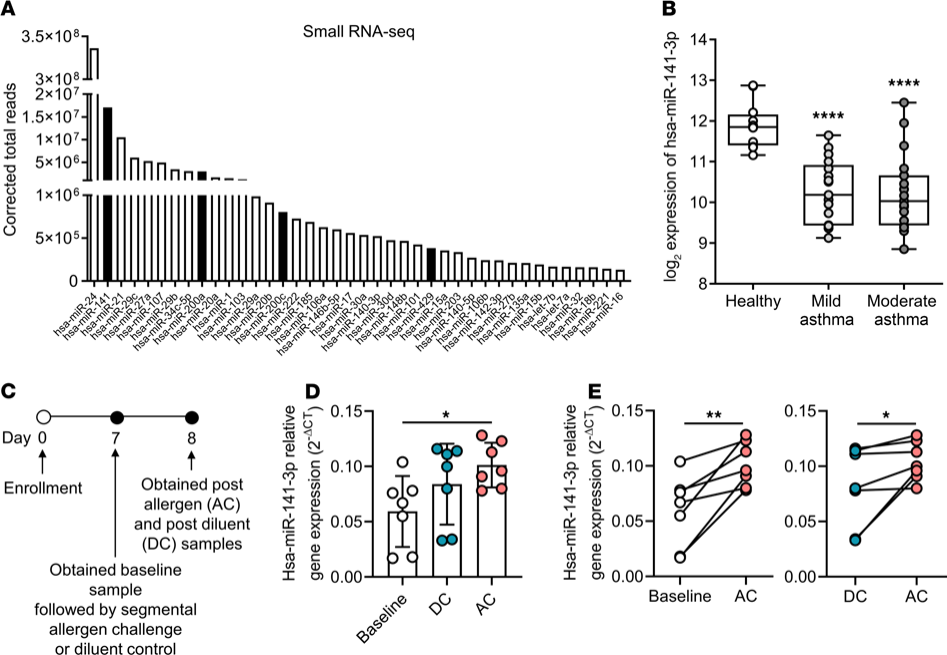
miR-141 is abundantly expressed in the human airway epithelium and induced upon airway allergen challenge in asthma. (**A**) Forty most highly expressed miRNAs by small RNA sequencing analysis of bronchial epithelial brushings (*n* = 16). miR-141/200 family miRNAs (141/200a/200c/429) are highlighted by black bars. (**B**) Microarray analysis of hsa-miR-141-3p in human epithelial brushings from mild asthmatics (not using inhaled corticosteroids) and moderate asthmatics (using inhaled corticosteroids) compared with healthy controls (*n* = 12–16/group, 1-way ANOVA with Dunnett’s multiple comparison test, ****P < 0.0001). (**C**) Timeline of segmental airway allergen challenge of allergic asthmatic subjects for collection of bronchial brushings. (**D** and **E**) Expression level of hsa-miR-141-3p by TaqMan qPCR in bronchial brushings collected at baseline and 1 day following allergen challenge (AC) or diluent control (DC) demonstrated by group (**D**) and paired analysis (**E**) (*n* = 7/group, 1-way ANOVA followed by Dunnett’s multiple comparison test, *P < 0.05, in **D**; 2-tailed paired *t* test; *P < 0.05, **P < 0.01 in **E**).

We performed miRNA profiling by small RNA sequencing (RNA-seq) of human bronchial epithelial brushings and found that hsa-miR-141-3p was the second most highly expressed miRNA in the airway epithelium (Figure 1A). Including hsa-miR-141-3p, 4 members of the miR-141/200 family (miR-141/200a/200c/429) were among the top 40 most abundantly expressed miRNAs in bronchial epithelial brushings. We have previously reported decreased hsa-miR-141-3p expression in bronchial epithelial brushings from 16 mild asthmatic subjects (not using inhaled corticosteroids) and 12 healthy controls using miRNA microarray data (16). In additional analyses, we found that hsa-miR-141-3p is repressed in moderate asthmatic subjects (using inhaled corticosteroids), as well (Figure 1B). We examined the expression of miR-141 further in 7 allergic asthmatic subjects who underwent bronchoscopy with collection of bronchial brushings before and after segmental allergen challenge or instillation of diluent control (Figure 1C; patient demographics provided in Supplemental Table 1; supplemental material available online with this article; https://doi.org/10.1172/jci.insight.139019DS1). Hsa-miR-141-3p expression, as measured by TaqMan qPCR, was induced in the epithelium 24 hours after the allergen challenge compared with baseline and diluent control samples (Figure 1, D and E). These results show that the miR-141/200 family, including hsa-miR-141-3p, is highly expressed in human airway epithelium ex vivo, and that miR-141 is modulated in the airway epithelium in asthma.

### CRISPR/Cas9 targeting of the MIR141 gene successfully decreases mature hsa-miR-141-3p expression in primary HBECs.

**Figure 2:**
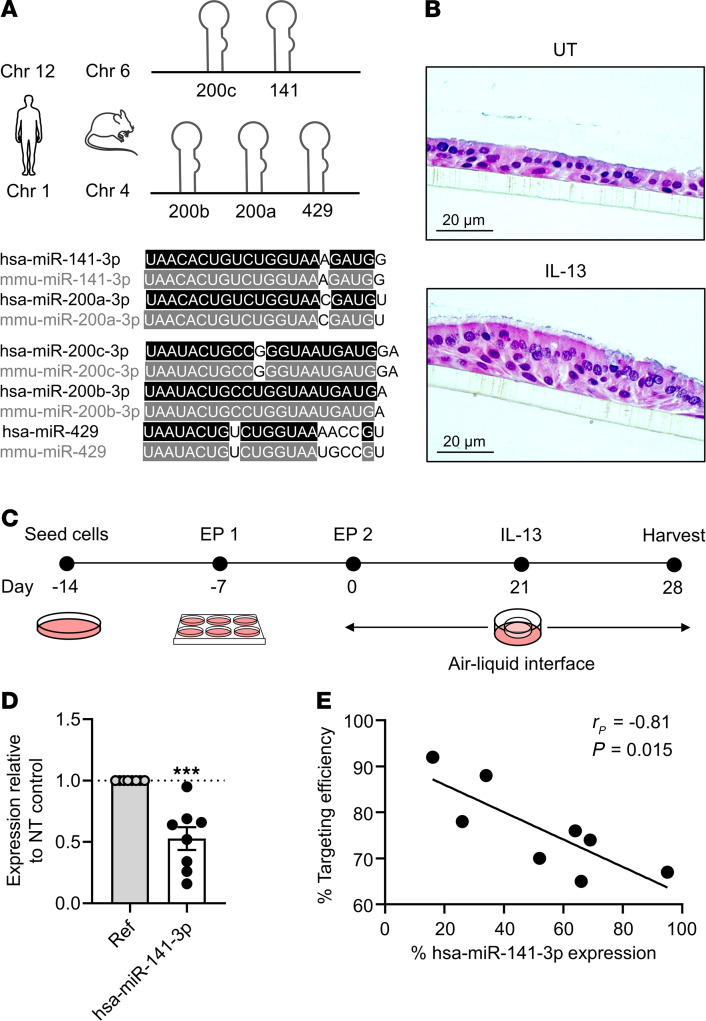
CRISPR/Cas9-mediated knockdown of miR-141 in primary HBECs grown at air-liquid interface. (**A**) Mature miRNA sequences and genomic location of the miR-141/200 family in humans and mice. (**B**) Representative H&E-stained filter sections of human bronchial epithelial cells (HBECs) cultured at air-liquid-interface (ALI) under untreated (UT) conditions or IL-13 stimulation, harvested on day 28 (representative of 4 unique donors). Scale bar: 20 μm. (**C**) Electroporation-based (EP-based) CRISPR/Cas9 protocol established in an in vitro ALI system (days 0–28, ± IL-13 on days 21–28) using HBECs. (**D**) Expression level of hsa-miR-141-3p by TaqMan qPCR following administration of *MIR141*-targeting versus nontargeting (NT) gRNAs normalized to reference miRNAs hsa-miR-103a-3p and hsa-miR-191-5p (Ref) (*n* = 8, 2-tailed *t* test; ***P < 0.001). (**E**) Correlation of *MIR141*-targeting efficiency score assessed by Sanger DNA sequencing and ICE Synthego analysis and hsa-miR-141-3p expression levels by qPCR. *r_P_*, Pearson correlation coefficient.

To study the role of miR-141 in the airway epithelium, we developed an electroporation-based dual guide RNA (gRNA; cRNA:tracrRNA) CRISPR protocol that enabled *MIR141* gene repression in HBECs grown in monolayer cultures. All 5 family members of the miR-141/200 family are shown in Figure 2A. Subsequent transfer to air-liquid-interface (ALI) generated a fully differentiated airway epithelium (representative sections in Figure 2B; timeline outlined in Figure 2C). On day 28, we harvested HBECs that received either *MIR141* gene-targeting gRNA or nontargeting (NT) gRNA control and isolated DNA to confirm editing efficiency by Sanger sequencing (*MIR141* gRNA targeting sites are outlined in Supplemental Figure 1). Across 9 unique HBEC donors, we estimated targeting efficiency of *MIR141* knockdown to be 65%–95% (Supplemental Figure 2A). The expression of mature hsa-miR-141-3p was significantly reduced upon *MIR141* targeting compared with the NT control (Figure 2D). Expression of other miR-141/200 family miRNAs in *MIR141*-targeted HBECs was not significantly reduced compared with NT control (Supplemental Figure 2B). Furthermore, repression of hsa-miR-141-3p expression in CRISPR/Cas9-targeted cells correlated significantly with the estimated targeting efficiency (Figure 2E).

### miR-141 repression reduces IL-13–induced mucus in primary HBECs.

**Figure 3:**
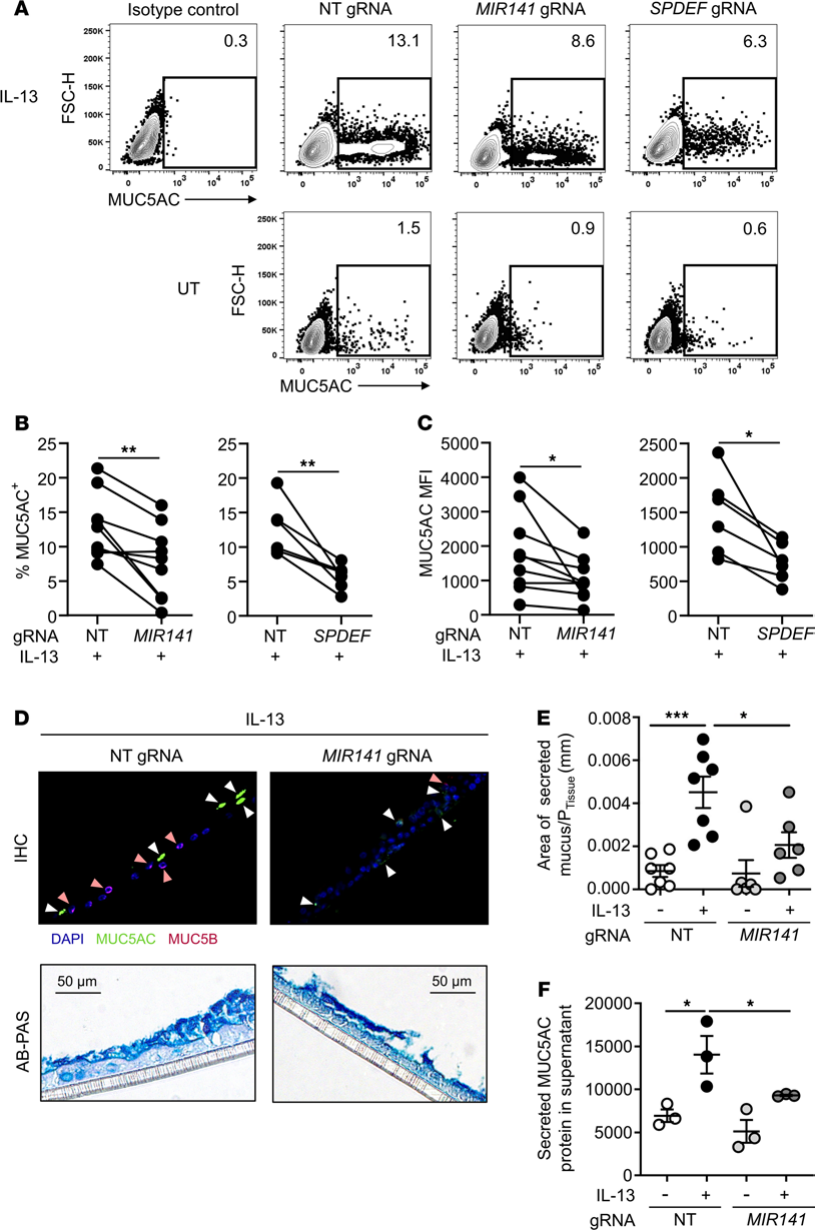
CRISPR/Cas9 targeting of miR-141 reduces IL-13–induced mucus. (**A**) Representative contour plots demonstrating MUC5AC^+^ cells in ALI-cultured human bronchial epithelial cells (HBECs) stimulated with (top panel) or without IL-13 (untreated controls; UT) (bottom panel) that have undergone gene editing with nontargeting (NT), *MIR141*, or *SPDEF* gRNAs. (**A**–**C**)Analysis was performed by intracellular flow cytometry (gated on forward scatter, FSC, singlets). Paired analysis of MUC5AC^+^ cells (% of all HBECs) (**B**) and MUC5AC mean fluorescent intensity (MFI) (**C**) (*n* = 9, 2-tailed paired *t* test, *P < 0.05, **P < 0.01). (**D**) HBEC filter sections stained with fluorescent antibodies for MUC5AC and MUC5B (top panel, positive staining indicated by white and pink arrows, respectively) and Alcian Blue-Periodic Acid Schiff (AB-PAS) (bottom panel). Scale bar: 50 μm. (**E** and **F**) Quantification of mucus-producing cells in AB-PAS–stained HBEC filter sections (**E**) and secreted MUC5AC assessed by dot blot analysis of apical wash (**F**) from ALI-cultured HBECs following NT or *MIR141* gRNA delivery (*n* = 3–7/group, 1-way ANOVA followed by the Holm-Sidak test; *P < 0.05, ***P < 0.001).

We next studied the effect on the IL-13–inducible airway mucin MUC5AC following *MIR141* gene editing in primary HBECs. Using intracellular flow cytometry, we found that *MIR141* targeting significantly decreased the frequency of MUC5AC-expressing cells following IL-13 stimulation compared with NT gRNA control HBEC cultures (Figure 3, A and B). CRISPR/Cas9 targeting of the goblet cell transcription factor *SPDEF* also resulted in significantly decreased MUC5AC^+^ cells, as recently shown (21). Gene editing reduced both the frequency of MUC5AC-expressing cells and mean fluorescence intensity (MFI), reflecting the amount of MUC5AC-binding antibodies, in *MIR141* and *SPDEF*-targeted HBECs compared with HBECs that received the NT gRNA control (Figure 3C). Decreased MUC5AC expression was also apparent in immunofluorescent staining of *MIR141*-targeted IL-13–stimulated HBECs in filter sections (Figure 3D). MUC5B was weakly detected in both the NT gRNA control and *MIR141*-targeted cells. Image analysis of Alcian Blue–Periodic Acid Schiff–stained (AB-PAS–stained) filters revealed a significant reduction of the area of secreted mucus in *MIR141*-targeted HBECs compared with NT control HBECs under IL-13–stimulated conditions (Figure 3E). In addition, we quantified secreted MUC5AC protein by dot blot analysis of apical wash samples collected from IL-13–stimulated or untreated ALI cultures (Figure 3F). The results confirmed a significant decrease of MUC5AC following *MIR141* gene editing when compared with NT gRNA controls following IL-13 stimulation. These results indicate that miR-141 regulates IL-13–induced MUC5AC production by epithelial cells.

### Epithelial miR-141 repression is associated with a reduction in mucus-producing goblet cell numbers.

**Figure 4:**
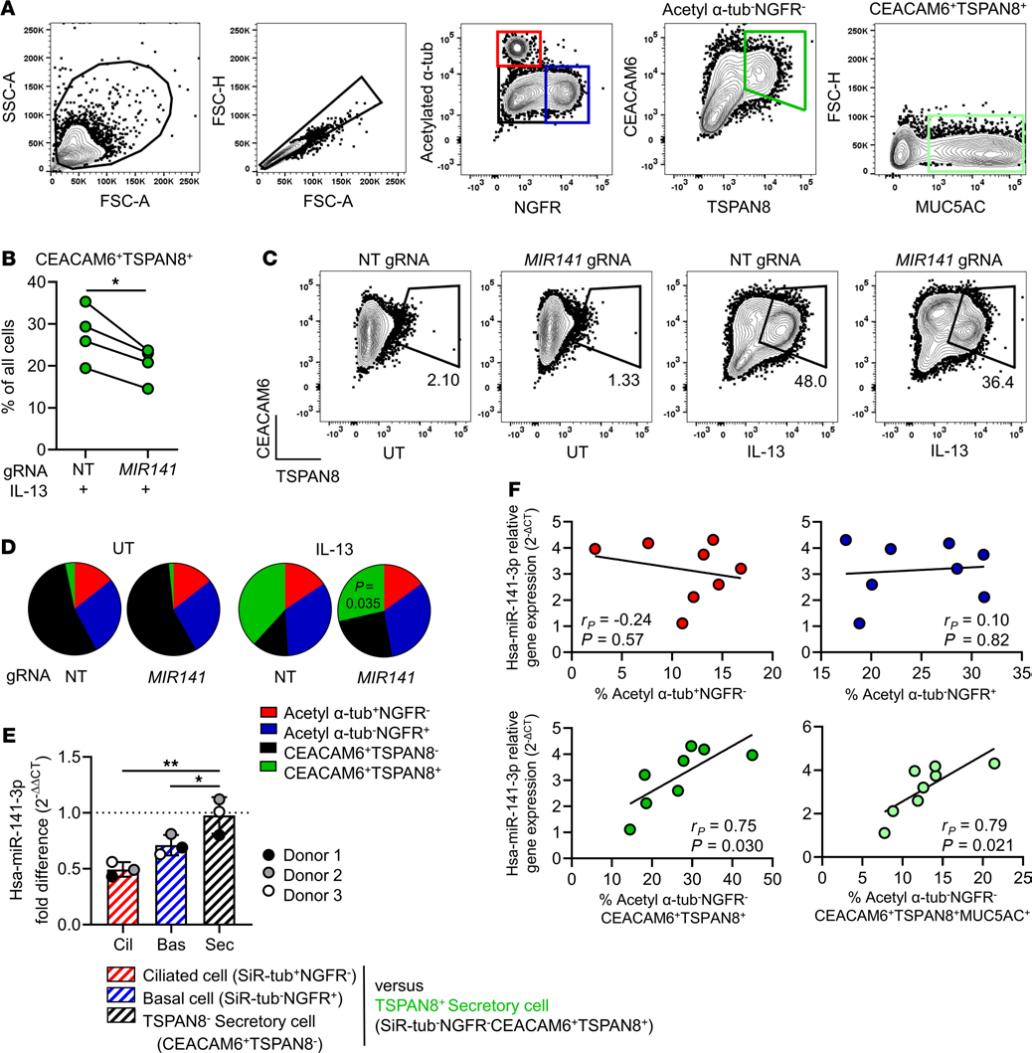
miR-141 repression in gene-edited airway epithelial cells is associated with a reduction in mucus-producing goblet cell numbers. (**A**) Flow cytometry gating strategy of acetylated α-tubulin^+^NGFR^–^ (red gate, ciliated cells), acetylated α-tubulin^–^NGFR^+^ (blue gate, basal cells), acetylated α-tubulin^–^NGFR^–^CEACAM6^+^TSPAN8^+^ (green gate, secretory cells), and acetylated α-tubulin^–^NGFR^–^CEACAM6^+^TSPAN8^+^MUC5AC^+^ (light green gate, MUC5AC^+^ secretory cells) cells. (**B**) Frequency of acetylated α-tubulin^–^NGFR^–^CEACAM6^+^TSPAN8^+^ secretory cells (% of all cells, *n* = 4/group) in human bronchial epithelial cell (HBEC) cultures that have undergone gene editing with nontargeting (NT) or *MIR141*-targeting gRNAs, subsequently grown at air-liquid-interface (ALI) with IL-13 stimulation (2-tailed paired *t* test). (**C**) Representative contour plots demonstrating CEACAM6^+^TSPAN8^+^ secretory cells in ALI cultures of untreated (UT, left) or IL-13–stimulated (right) HBECs after gene editing with NT or *MIR141* gRNAs. (**D**) Frequency of ciliated cells (red), basal cells (blue), TSPAN8^–^ secretory cells (black), and TSPAN8^+^ secretory cells (green) (% of all cells, *n* = 4/group) in ALI-cultured NT or *MIR141*-targeted HBECs with (IL-13) or without (UT) IL-13 (2-tailed paired *t* test). (**E**) Hsa-miR-141-3p expression fold difference assessed by TaqMan qPCR in FAC-sorted ciliated cells (SiR-tubulin^+^NGFR^–^), basal cells (SiR-tubulin^–^NGFR^+^), and TSPAN8^–^ secretory cells compared with IL-13–inducible TSPAN8^+^ secretory cells (1-way ANOVA followed by Dunnett’s test; *P < 0.05, **P < 0.01). (**F**) Hsa-miR-141-3p expression levels assessed by TaqMan qPCR in relation to frequency of red, blue, green, or light green cell gates (as in **A**). *r_P_*, Pearson correlation coefficient.

To investigate the mechanism by which miR-141 regulates MUC5AC, we measured specific changes in airway epithelial subpopulations in response to *MIR141* targeting by flow cytometry. We analyzed airway epithelial subpopulations using a panel of antibodies targeting subset-specific cellular markers, including basal (nerve growth factor receptor; NGFR) and ciliated (acetylated α-tubulin) cell markers that have been described previously (22) and newly identified markers by single cell RNA-seq (scRNA-seq) analysis of human bronchial brushings (23) (Figure 4A). *MIR141* gene editing in IL-13–stimulated HBECs resulted in significantly lower frequency of Tetraspanin 8 (TSPAN8^+^) secretory cells (defined as acetylated α-tubulin^–^NGFR^–^CEACAM6^+^TSPAN8^+^; CEA Cell Adhesion Molecule 6 [CEACAM6]) compared with IL-13–stimulated NT gRNA control HBECs (Figure 4B). TSPAN8^+^ secretory cells were only present in IL-13–stimulated cultures (Figure 4C) and were the major MUC5AC-producing population detected by intracellular MUC5AC staining. The proportion of ciliated, basal, and secretory cells was similar in *MIR141*-targeted and NT gRNA control HBEC cultures under untreated conditions (Figure 4D).

To assess subset-specific expression of miR-141, we used nonpermeabilized cells and a modified staining panel. The intracellular antibody to acetylated α-tubulin was replaced with SiR-tubilin, a live cell permeable microtubule dye, and fresh IL-13–stimulated ALI-cultured HBECs from 3 unique donors were FACS purified. We found that hsa-miR-141-3p was enriched in TSPAN8^+^ secretory cells (SiR-tubulin^–^NGFR^–^CEACAM6^+^) compared with ciliated cells (SiR-tubulin^+^NGFR^–^) and basal cells (SiR-tubulin^–^NGFR^+^) (Figure 4E). Additionally, the expression level of hsa-miR-141-3p was similar in TSPAN8^+^ and TSPAN8^–^ secretory cells, where TSPAN8^–^ cells are likely to be precursors of TSPAN8^+^ secretory cells. These results suggest that a reduced level of miR-141 in CRISPR/Cas9-targeted cultures affects secretory/goblet cells. Further analysis revealed that the increased number of TSPAN8^+^ secretory cells and MUC5AC^+^ goblet cells (defined as acetylated α-tubulin^–^NGFR^–^CEACAM6^+^TSPAN8^+^MUC5AC^+^) significantly correlated with hsa-miR-141-3p expression levels in IL-13–stimulated HBEC cultures (Figure 4F), consistent with the finding that hsa-miR-141-3p is enriched in FACS-sorted secretory cells. Ciliated cells (acetylated α-tubulin^+^NGFR^–^) and basal cells (acetylated α-tubulin^–^NGFR^+^) displayed no significant modulation in relation to hsa-miR-141-3p expression, suggesting that miR-141 targets genes that specifically regulate secretory/goblet cells. Moreover, analysis of other miR-141/200 family miRNAs revealed significant correlations between increasing frequency of TSPAN8^+^ secretory cells in IL-13–stimulated HBECs and expression levels of hsa-miR-200b-3p, hsa-miR-200c-3p, and hsa-miR-429 (Supplemental Figure 3). No other airway epithelial subpopulations correlated with the expression of miR-200b/c/429, including MUC5AC^+^ goblet cells, which only demonstrated a significant association to hsa-miR-141-3p expression.

### MIR141 targeting of epithelial cells interferes with the response to IL-13 and results in reduced expression of goblet cell genes.

**Figure 5:**
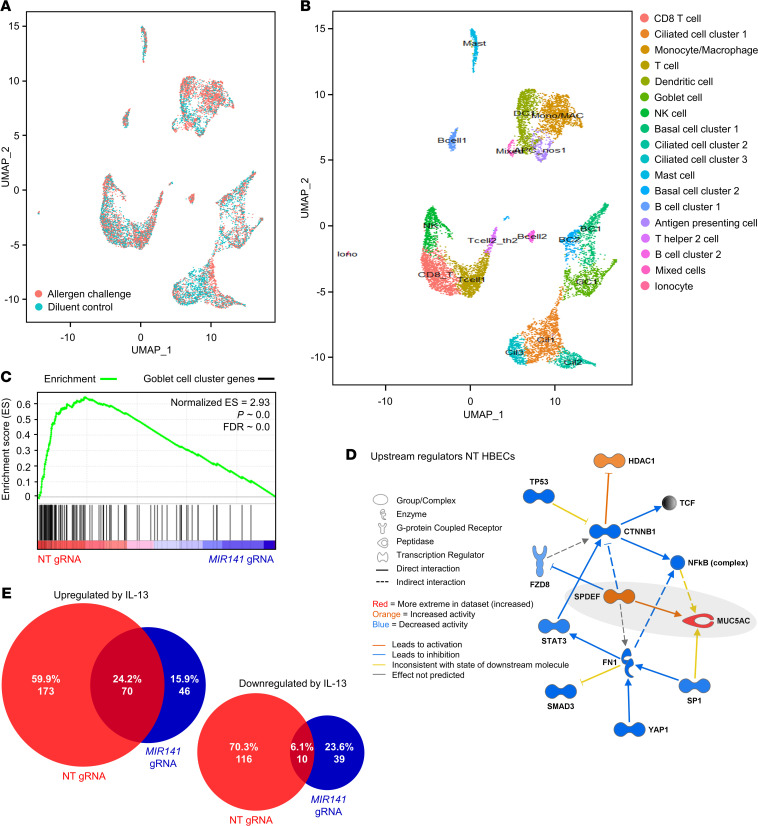
*MIR141* targeting interferes with IL-13 signaling and results in reduced expression of goblet cell genes. Single cell RNA sequencing analysis of bronchial epithelial brushings obtained from allergic asthmatic subjects 24 hours after segmental allergen challenge (*n* = 4) or diluent control (*n* = 4). (**A**) Overlay of cellular clusters from allergen challenge and diluent control. (**B**) Eighteen identified cellular gene clusters with annotations. (**C**) Gene set enrichment analysis of 100 goblet cell cluster genes (Supplemental Table 3) in human bronchial epithelial cells (HBECs) that have undergone gene editing with nontargeting (NT) or *MIR141* gRNAs, subsequently grown at air-liquid-interface with IL-13 stimulation (*n* = 4/group). (**D**) Upstream regulator SPDEF (*P* = 2.5 × 10^–12^) identified by Ingenuity Pathway Analysis (IPA) of genes differentially expressed (FDR < 0.1) in NT HBECs following IL-13 stimulation. (**E**) Overlapping and uniquely expressed genes (IL-13 versus untreated) in NT and *MIR141* gRNA HBEC cultures (*n* = 3–4/group) assessed by RNA sequencing (χ^2^ test; ****P < 0.0001).

To study the consequences of miR-141 repression in epithelial cells on a transcriptional level, we analyzed *MIR141*-targeted HBECs and NT gRNA control HBECs by RNA-seq. HBEC cultures with the most efficient repression of miR-141-3p expression (48%–84% reduction compared with NT controls) and CRISPR-targeting efficiency (65%–95% by Sanger sequencing) were selected for the analysis. A goblet cell gene signature was generated by scRNA-seq analysis of bronchial epithelial brushings from 4 allergic asthmatic individuals that were collected 24 hours following segmental allergen challenge or diluent control (patient demographics in Supplemental Table 2). Cellular clusters in epithelial brushings after allergen challenge demonstrated a large overlap with diluent control samples (Figure 5A), and a total of 18 distinct cellular clusters was defined (Figure 5B). The cluster analysis identified a signature of 100 genes (Supplemental Table 3) that was significantly enriched in goblet cells and included well-known goblet cell genes such as the mucins *MUC5AC, MUC5B, MUC1,* and Secretoglobin Family 1A Member 1 (*SCGB1A1*), Trefoil Factor 3 (*TFF3*), *CEACAM6*, and *SPDEF*. The 100–goblet cell gene signature was analyzed across 4 NT control HBEC donors and 4 *MIR141*-targeted HBEC donors using Gene Set Enrichment Analysis (GSEA). In bulk RNA-seq analysis, this goblet cell gene signature was highly enriched in the IL-13–stimulated NT gRNA control condition compared with IL-13–stimulated *MIR141*-targeted cells (Figure 5C), supporting our findings of decreased goblet cell frequency in *MIR141*-targeted HBECs by flow cytometry. IL-13–stimulated NT gRNA control HBECs also exhibited a significant enrichment of ciliated cell genes (Supplemental Figure 4 and Supplemental Table 3); however, the goblet cell gene signature displayed the highest enrichment score. Furthermore, analysis of IL-13–induced changes in global gene expression using Ingenuity Pathway Analysis (IPA) identified the goblet cell transcription factor SPDEF to be the most likely upstream regulator of the transcriptional changes induced by IL-13 in NT gRNA control HBECs (Figure 5D). The SPDEF-network had activation *Z* score of 3.96 and demonstrated a significant overlap with the RNA-seq data set (*P* = 2.5 × 10^–12^) where the expression of 21 of 27 genes downstream of SPDEF was consistent with activation of SPDEF. In contrast, IPA of differentially expressed genes in IL-13–stimulated *MIR141*-targeted cells revealed a complete lack of goblet cell–related networks. Indeed, in response to IL-13 stimulation, a significant number of genes was differentially expressed in NT gRNA control HBECs (both upregulated and downregulated) but not in MIR141-targeted HBECs (Figure 5E), which may suggest that miR-141 expression is required for a normal epithelial response to IL-13 and goblet cell development.

### miR-141 repression leads to increased basal cell gene expression.

**Figure 6:**
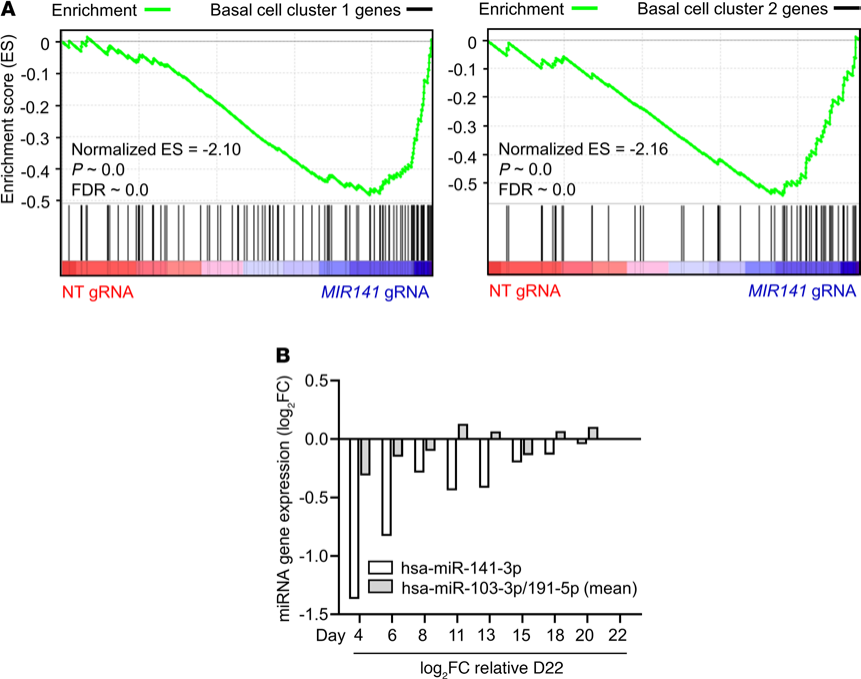
*MIR141*-targeted HBECs demonstrate increased basal cell gene expression. (**A**) Gene Set Enrichment Analysis of basal cell genes (100 genes, basal cell cluster 1; 62 genes, basal cell cluster 2) in human bronchial epithelial cells (HBECs) that have undergone gene editing with nontargeting (NT) or *MIR141* gRNAs, subsequently grown at air-liquid-interface (ALI) with IL-13 stimulation (*n* = 4/group). Basal cell clusters were identified by single cell RNA sequencing analysis of bronchial epithelial brushings obtained from allergic asthmatic subjects (*n* = 4) (Supplemental Table 3). (**B**) Hsa-miR-141-3p expression levels in ALI-cultured HBEC cultures from day 4 (confluent) to day 22 (differentiated) assessed by microarray (*n* = 2 samples/time point).

Basal cells of the airway epithelium give rise to multiple cell lineages, including mucus-producing goblet cells (24). To learn more about how miR-141 disruption interferes with responses of mucus secretory cells, we included the basal cell gene signatures obtained from scRNA-seq of bronchial brushings (Figure 5B and Supplemental Table 3) in the GSEA analysis of *MIR141*-targeted and NT gRNA control HBECs. We found that *MIR141*-targeted HBECs exhibited a significant enrichment of basal cell genes compared with NT gRNA controls (Figure 6A). Next, we studied the expression of miR-141, as assessed by microarray, in differentiating ALI cultures every 2–3 days from confluent cultures on day 4 to a fully differentiated epithelium on day 22. Hsa-miR-141-3p exhibited a dynamic expression pattern, with the lowest expression at day 4 and stepwise increases reaching a peak on day 22 (Figure 6B). These findings suggested that *MIR141* may be involved in the transition of basal cells into mucus secretory goblet cells that occurs in human trachea and ALI cultures (24).

### Large numbers of predicted and confirmed miR-141 targets are expressed during basal-to-mucus secretory cell transition.

**Figure 7:**
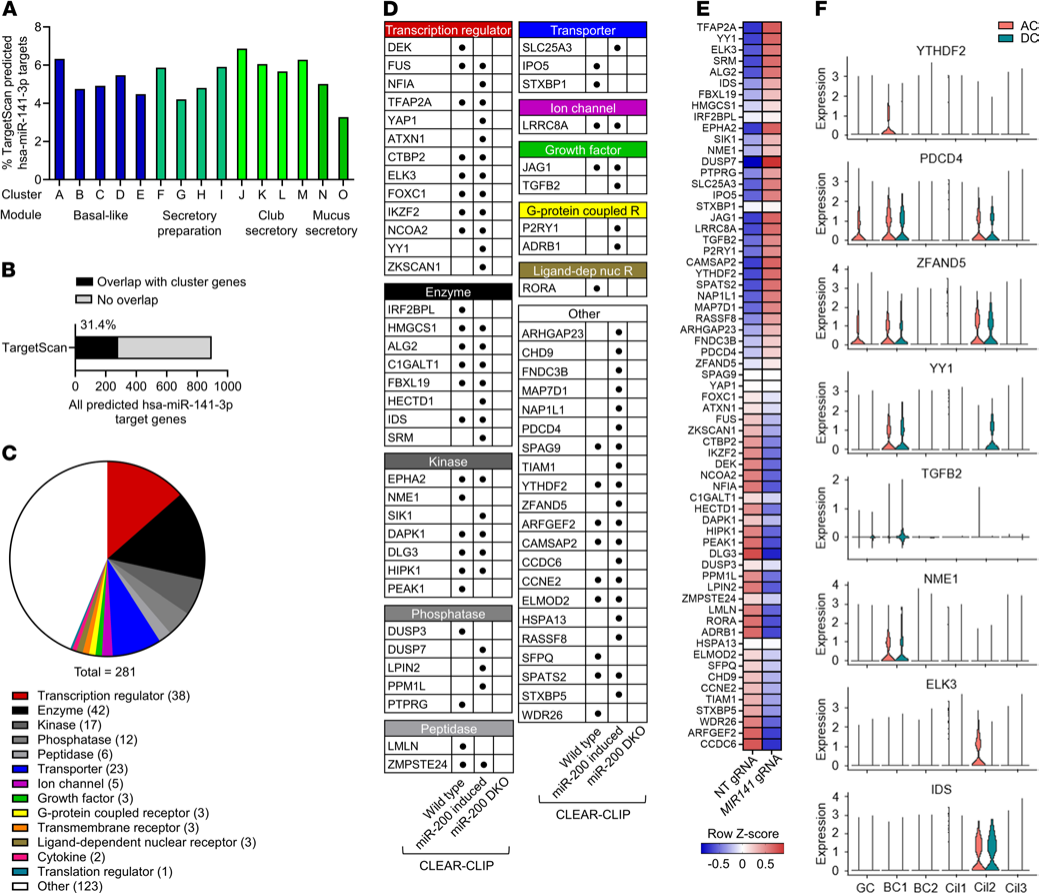
miR-141-3p targets a large number of genes expressed during the transition from basal cell to mucus secretory cell. (**A**) Percentage TargetScan predicted hsa-miR-141-3p targets in distinct clusters identified by pseudotime gene expression analysis of epithelial cell differentiation from basal-like presecretory cells into mucus secretory cells in human airways. Pseudotime gene expression analysis was published by Goldfarbmuren et al. (24). (**B**) Percent predicted targets in pseudotime gene clusters of all TargetScan-predicted hsa-miR-141-3p targets. (**C**) Type of protein encoded by 281 TargetScan-predicted hsa-miR-141-3p gene targets found in pseudotime clusters. (**D**) Sixty-five TargetScan-predicted miR-141-3p gene targets found in pseudotime clusters and identified by differential CLEAR-CLIP analysis of primary murine epithelial cells from WT, miR-200 family–induced, and miR-200 family–double deficient mice (*n* = 3/group) (26). (**E**) Expression by RNA sequencing of 65 miR-141-3p target genes in HBECs that have undergone gene editing with NT or *MIR141* gRNAs, subsequently grown at ALI with IL-13 stimulation (*n* = 4/group). (**F**) Gene expression, as assessed by single cell RNA sequencing, of bronchial epithelial brushings obtained from allergic asthmatic subjects 24 hours after segmental allergen challenge (*n* = 4) or diluent control (*n* = 4).

Next, we compared the basal and goblet cell signatures derived from scRNA-seq (Supplemental Table 3) with 4 distinct transitional states from basal-like cells to fully competent MUC5AC-expressing mucus secretory cells that were previously defined by pseudotime gene cluster analysis of an independent data set (24). Using TargetScan v7.2, we analyzed the frequency of genes in 15 transitional gene clusters belonging to the 4 different cell states (basal-like, secretory preparation, club secretory, mucus secretory) with a predicted conserved hsa-miR-141-3p seedmatch in the 3′UTR (25). A large number of predicted miR-141 targets (281 genes, 31.4% of all hsa-miR-141-3p predicted targets) overlapped with genes across the clusters (Figure 7, A and B). The largest defined groups of the 281 predicted target genes encoded enzymes and transcriptional regulators (Figure 7C). To increase the confidence of miR-141 targets identified during basal-to-secretory cell differentiation, we mined a recently published CLEAR-CLIP data set that captured individual miRNAs and their targeted RNA sites in WT, miR-200 family–induced, and miR-200 family–deficient murine epithelial cells (26). Almost all 281 genes had a miR-141-3p binding site that was conserved in the murine genome. Using differential CLEAR-CLIP peaks, we confirmed 65 genes to be experimentally captured in WT or miR-200–induced epithelial cells but not in miR-200 family–deficient cells (Figure 7D). Differential RNA-seq analysis of the 65 experimentally confirmed target genes in IL-13–stimulated *MIR141*-targeted and NT control HBECs revealed a significant number of derepressed miR-141-3p targets in *MIR141*–gene edited HBECs (Figure 7E), indicating that miR-141 may regulate a network of genes that are repressed during normal goblet cell differentiation. Most derepressed genes belonged to early basal-like and secretory preparation clusters (Supplemental Figure 5A). Network analysis of all 31 derepressed miR-141-3p target genes by IPA highlighted both inhibitory and activating pathways to MUC5AC, but the net effect predicted inhibition of MUC5AC in the network (Supplemental Figure 5B), which is consistent with our observations of decreased MUC5AC expression in *MIR141*-targeted cultures. Some miR-141 target genes that were derepressed in *MIR141*-targeted HBECs were broadly detected by scRNA-seq in goblet, basal, and ciliated cells from asthmatic airways (Supplemental Figure 6). In addition, several miR-141 target genes were selectively expressed in goblet, basal, or ciliated cells, and some genes were differentially expressed following segmental allergen challenge compared with diluent controls (Figure 7F). For instance, allergen challenge induced programmed cell death 4 (*PDCD4*) expression in goblet cells. Depletion of PDCD4 by RNA interference in vivo via intranasal administration suppresses airway mucus secretion in OVA-induced allergic airway inflammation (27).

### Inhibition of mmu-miR-141-3p in vivo improves allergen-induced airway hyperresponsiveness.

**Figure 8:**
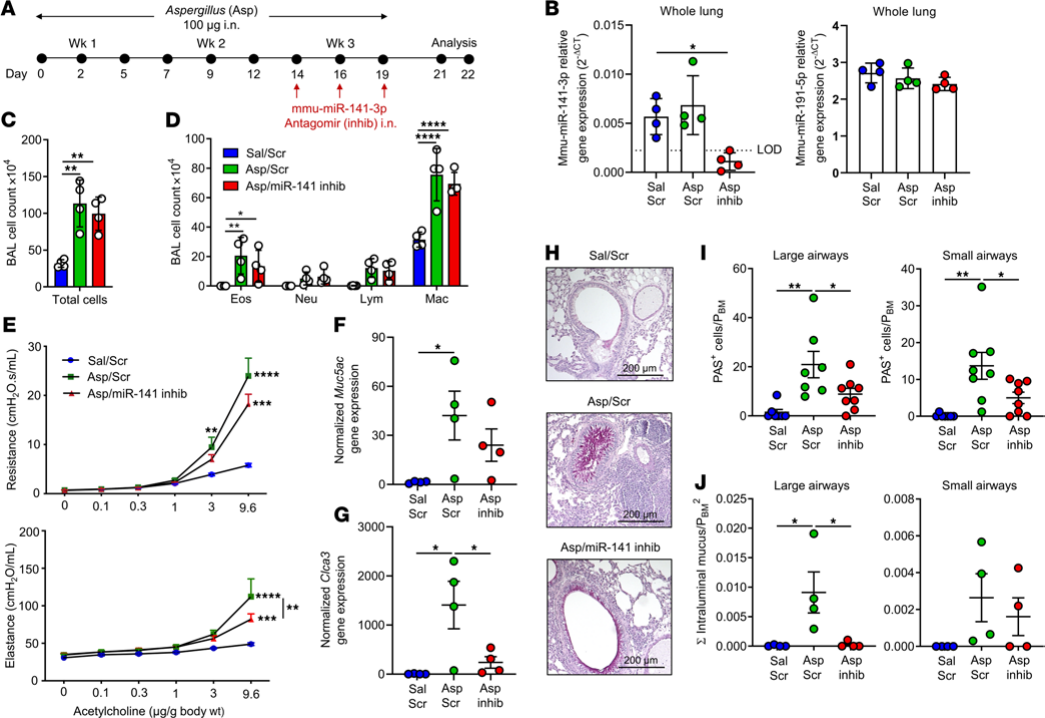
Blockade of mmu-miR-141-3p improves airway hyperresponsiveness and decreases secreted mucus in an experimental mouse model of asthma. (**A**) Timeline of allergen-induced model of asthma induced by intranasal (i.n.) exposure to fungal allergen *Aspergillus fumigatus* (Asp) 3 times per week for 3 weeks; details are provided in the Methods section. (**B**) Expression level of mmu-miR-141-3p (left) and nontargeting control mmu-miR-191-5p (right) in whole lung tissue by TaqMan qPCR normalized to reference miRNA mmu-miR-103-3p (*n* = 4, 1-way ANOVA followed by Tukey test; *P < 0.05). (**C** and **D**) Total cells (**C**) and cellular distribution (**D**) in bronchoalveolar lavage (BAL) obtained from mice exposed to Asp in combination with mmu-miR-141-3p antagomir (Asp/miR-141 inhib), Asp in combination with scrambled antagomir (Asp/Scr), and sterile saline in combination with Scr antagomir (Sal/Scr) (*n* = 7-8/group, 1-way (**C**) or 2-way (**D**) ANOVA followed by Tukey test; *P < 0.05, **P < 0.01, ****P < 0.0001). Eos, eosinophils; Neu, neutrophils; Lym, lymphocytes; Mac, macrophages. (**E**) Total respiratory system resistance and elastance measured in mice exposed to Asp/miR-141 inhib, Asp/Scr, and Sal/Scr (*n* = 7–8/group, repeated measures 2-way ANOVA followed by Bonferroni correction; **P < 0.01, ***P < 0.001, ****P < 0.0001). (**F** and **G**) Gene expression of *Muc5ac* (**F**) and *Clca1* (**G**) assessed by qPCR analysis of lung tissue homogenate 72 hours after the final allergen challenge (*n* = 4/group, 1-way ANOVA followed by the Tukey test; *P < 0.05). (**H**) Representative Alcian Blue–Periodic Acid Schiff–stained (AB-PAS–stained) lung sections from Asp/miR-141 inhib, Asp/Scr, and Sal/Scr mice (representative of 4 mice). (**I** and **J**) Quantification of PAS^+^ cells per perimeter of basal membrane (**I**) and intraluminal mucus per basal membrane area (**J**) in large (>0.80 mm) and small (<0.80 mm) airways (*n* = 7–8/group, 1-way ANOVA followed by the Tukey test; *P < 0.05, **P < 0.01).

Previous studies in mice have demonstrated highly efficient epithelial uptake of antagomirs administered directly to the airways (28). To study the effects of miR-141 inhibition on mucus regulation in vivo, we used a model of allergic asthma in which we challenged mice intranasally with the fungal allergen *Aspergillus fumigatus* (Figure 8A) or administered sterile saline as a sham challenge control. The expression level of mmu-miR-141-3p in the lung tissue was significantly reduced upon administration of the miR-141-3p–targeting antagomir compared with the NT scramble antagomir (Figure 8B). Analysis of cellular populations in bronchoalveolar lavage (BAL) revealed that the composition of inflammatory cells was unaffected by mmu-miR-141-3p antagomir treatment (Figure 8, C and D). Airway hyperresponsiveness was measured 48–72 hours after the final allergen challenge, and we found that *Aspergillus*-challenged mice that received the mmu-miR-141-3p antagomir were less reactive to acetylcholine compared with *Aspergillus*-challenged mice that received the scrambled antagomir, as assessed by total respiratory system resistance and elastance (Figure 8E). Furthermore, *MUC5AC* gene expression in whole lung homogenate displayed a decreasing trend in mice that received mmu-miR-141-3p antagomir (Figure 8F), and the goblet cell–specific gene *CLCA1* was significantly downregulated upon mmu-miR-141-3p antagomir treatment in allergen-challenged lungs (Figure 8G), suggesting that mmu-miR-141-3p inhibition blocked allergen-induced goblet cell differentiation.

### Inhibition of mmu-miR-141-3p decreases epithelial mucus induction in vivo.

We prepared lung sections from *Aspergillus*-challenged mice to examine mucus-producing cells in the lung tissue (representative AB-PAS–stained sections in Figure 8H). Mice that were challenged with airway allergen and that received mmu-miR-141-3p antagomir had significantly decreased numbers of mucus-expressing epithelial cells in the large and small airways (Figure 8I). Furthermore, large airways also exhibited a decrease in secreted mucus in the airway lumen (Figure 8J) after mmu-miR-141-3p antagomir treatment compared with scramble antagomir treatment, demonstrating that inhibition of mmu-miR-141-3p has a therapeutic effect in reducing key features of allergen-induced asthma.

## Discussion

Although the study of miRNAs in asthma and allergic inflammation is a relatively young field, it is evident that these conditions are accompanied by changes in miRNA expression (16, 29–31), which, in turn, promote disease pathology (17, 28, 32, 33). However, a tremendous amount of work remains to assign physiological functions to individual miRNAs and to integrate them in our understanding of cellular mechanisms in specific pathological contexts. In the current study, we found that miR-141 regulates the increase in airway epithelial goblet cell numbers, goblet cell MUC5AC gene and protein expression, and epithelial mucus production that occur after stimulation with IL-13. MUC5AC is a major component of the pathological mucus gel in T2-high asthma (12), a condition associated with increased IL-13 (7, 34), and mucus plugging is a feature of both severe chronic T2-high asthma and of severe asthma exacerbations (3, 4, 35, 36). To determine whether inhibition of miR-141 could represent a therapeutic strategy in asthma, we studied the effect in a murine model of allergic asthma and observed that intranasal administration of an antagomir specific to mmu-miR-141-3p reduces airway hyperresponsiveness and mucus production without altering cellular inflammation, suggesting a direct effect on epithelial cells and not by indirect inflammatory cell signaling. These data suggest that inhibition of miR-141 could provide a novel strategy for treatment of pathological mucus production and airflow obstruction in T2-high asthma.

Although our studies of patients with asthma show lower miR-141 levels in the airway epithelium in asthma than in health during a stable phase of disease, our challenge study shows induction of miR-141 shortly after allergen exposure in allergic asthmatics. This suggests that miR-141 may play a role in regulating epithelial differentiation in response to allergens. A recent study of chronic rhinosinusitis, another allergic disease driven by IL-13, found that the nasal epithelium displayed basal cell hyperplasia where mucus secretory and ciliated cells got trapped in an undifferentiated state, resulting in reduced epithelial diversity (37). The transcriptional changes caused by the allergic milieu persisted even in the absence of an allergic stimulus and were described as basal cell memory. In addition, the higher frequency of basal cells resulted in an increased pool of IL-33 and thymic stromal lymphopoietin (TSLP), which are potent drivers of allergic inflammation (38, 39). In the current study, we found that *MIR141* targeting of IL-13–stimulated HBECs increased basal cell gene expression. We identified multiple, experimentally confirmed miR-141 targets in a gene network expressed early during basal-to-mucus secretory cell differentiation, many of which were derepressed in *MIR141*-targeted cells and differentially expressed in allergic asthmatic airways following segmental allergen challenge. The initially low expression of miR-141 in differentiating ALI cultures would allow expression of basal cell genes that are targeted by miR-141, whereas subsequent increase of miR-141 in differentiating cells would suppress these genes to promote development of mucus secretory cells, a process known to be induced by allergens. Indeed, we found that miR-141 expression was enriched in FACS-sorted secretory cells compared with basal and ciliated cells, and the increased expression level of miR-141 correlated specifically with the frequency of goblet cell populations but not with nonmucus producing airway epithelial subpopulations in IL-13–stimulated HBEC cultures. Thus, our results suggest that the functional effect of miR-141 regulation may be restricted to specific subpopulations of the airway epithelium.

The miR-141/200 family is encoded in 2 genomic clusters, which give rise to 5 highly homologous mature miRNAs that are well conserved across species. A recent study investigating CRISPR/Cas9-based editing of miRNA clusters found that a mutation in one hairpin could affect the expression of a miRNA that resided in the other hairpin of the same cluster (40). This observation was thought to be due to changes in the tertiary structure of the primary miRNA transcript (pri-miRNA), leading to a differential expression of the mature miRNA. However, the authors also reported that mutations were well tolerated, provided they did not disrupt critical elements such as stem length, bulge position, and terminal loops (40). These findings are important, since they imply that CRISPR/Cas9 editing of miRNAs can affect processing of the hairpin in a dual manner — directly through sequence alteration and disruption of sequence motifs, or structurally through changes to the pri-miRNA — thus highlighting the complexity of CRISPR/Cas9 targeting of miRNAs. We focused specifically on miR-141 in this study and were able to significantly downregulate hsa-miR-141-3p expression with relative preservation of the expression of the other miR-141/200 family miRNAs. Importantly, miR-141 has an identical seed sequence to miR-200a such that one could expect miR-141 and miR-200a to play additive or synergistic roles in airway epithelial responses. However, our results clearly show that targeting miR-141 alone can repress IL-13–induced mucus production. The remaining miR-141/200 family members are less homologous to miR-141 but could potentially have additional overlapping and/or divergent effects. Indeed, a recent study of the miR-141/200 family found that the 2 subgroups (distinguished by the seed sequence 141/200a and 200b/200c/429) bound to largely distinct sites, and cross-seed recognition was rare (i.e., 141/200a binding of RNAs with a 200b/200c/429 seedmatch) (41). However, many genes were regulated by multiple family members sharing the same seed sequence, suggesting that the miR-141/200 family cooperates in target recognition (41). In our study, hsa-miR-200a-3p was the only miR-141/200 family member whose expression did not show any relationship to the frequency of goblet cells in IL-13–stimulated HBEC cultures. This suggests that miR-141 and miR-200a may not share functional overlap in mucus regulation or that the lower expressed miR-200a is not sufficient to compensate for reduced expression of miR-141.

A previous study demonstrated that overexpression of each of the individual miR-141/200 family miRNAs or overexpression of 1 of the 2 clusters blocked epithelial to mesenchymal transition (EMT) in an in vitro–based EMT assay using murine mammary gland epithelial cells (42). The authors found that this effect was mediated through miR-141/200 targeting of *ZEB1* and *ZEB2*, 2 transcriptional repressors of E-cadherin. Thus, E-cadherin was upregulated due to miR-141/200 overexpression (42). Interestingly, E-cadherin is an important junction protein that maintains airway epithelial barrier function in asthma. Indeed, a recent study in mice found that lung epithelial loss of E-cadherin leads to spontaneous progressive epithelial damage, which ultimately mimics the lung pathology observed in asthma with goblet cell metaplasia, mucus production, and eosinophil infiltration (43). Another recent study found that miR-141 targets *FOXA2* in cervical cancer where *FOXA2* silencing mimicked the tumorigenic effect of miR-141 overexpression in cervical cancer cells (18). In the context of mucus cell metaplasia, the FOXA2 transcription factor is thought to play an important role in the transition of club cells to a goblet cell phenotype. Several other transcription factors, in addition to FOXA2, coordinate this transcriptional program, and disruption of these key mediators leads to mucus cell metaplasia (44), where specific targeted deletion of FOXA2 in the airway epithelium of mice results in spontaneous mucus metaplasia (8). In the current study, we expand on the list of reported transcriptional regulators and other factors that are targeted by miR-141 and expressed during mucus secretory cell development.

The functional diversity of epithelial tissues is dictated by the composition of differentiated cell subsets. Here, we found that targeting *MIR141* altered airway epithelial subpopulations, but only in IL-13–stimulated conditions. IL-13 stimulation of ALI-cultured HBECs potently promotes mucus metaplasia, and several recent studies have provided a detailed view of the hierarchical lineage of airway epithelial cells (24, 45, 46). We used available data sets to identify a network of predicted and confirmed miR-141 target genes that are dynamically expressed by precursor populations of mucus secretory cells in vivo in human airways and in vitro in HBECs cultured at ALI (24). Basal cells also give rise to club cells that can, in turn, transdifferentiate into ciliated cells and goblet cells (45), and asthmatic lungs contain ciliated cells that coexpress a number of goblet genes, including MUC5AC (46). In this study, we found that *MIR141* targeting resulted in a significant difference in TSPAN8^+^ secretory cell frequency by flow cytometry but did not alter frequencies of ciliated and basal cells. However, RNA-seq analysis revealed that *MIR141*-repressed epithelial cells had an enrichment of basal cell genes, suggesting that additional specific basal cell markers may be required to identify phenotypic changes in the basal cell compartment by flow cytometry.

The specific repression of mucus secretory cells upon *MIR141* targeting can explain the reduction in secreted and intracellular mucus that we observed both in our in vitro system and in vivo model of allergic asthma. An inhaled antagomir-based therapeutic approach is attractive in this context for several reasons. miR-141 expression is largely restricted to the bronchial tree in mice (21) and, therefore, accessible to intranasal antagomir administration. Previous studies in mice have reported close to 100% uptake in the airway epithelium using intranasal delivery (28). Indeed, mmu-miR-141-3p inhibition in our study successfully decreased mmu-miR-141-3p expression in the lung and resulted in *CLCA1* gene downregulation, which suggests defective goblet cell differentiation in response to allergen challenges to the airway. This result highlights that modulation of miR-141 in the airways may have important beneficial effects in asthma.

In summary, our studies of airway epithelial mucus regulation have identified a miRNA that is differentially expressed in asthma and that, upon repression, downregulates epithelial mucus production in vivo and in vitro and reduces airway hyperresponsiveness. Given the pathogenic role that mucus overproduction and plugging plays in airflow obstruction in chronic severe asthma and in severe asthma exacerbations, and the lack of therapeutics that specifically target mucus production in the airway, miR-141 and/or its mRNA targets may be valuable therapeutic targets in T2-high asthma. Direct investigation of overlapping or divergent roles of other miR-141/200 family members to mucus production in asthma is an important future direction.

## Methods

### Primary HBEC cultures.

Cryopreserved primary hHBECs were thawed and seeded on human placental collagen-coated (HPC; MilliporeSigma) 10 cm petri dishes (3 × 10^6^ cells/dish) on day –14 (Figure 2B). The seeding medium (described in supplemental methods) was supplemented with Rho-associated protein kinase inhibitor Y-27632 (10 μM, ROCK inhibitor, Enzo Life Sciences) right before use (47). After 24 hours, the seeding medium was removed and cells were rinsed with PBS to exclude nonadherent dead cells; they were then propagated in complete BEGM (Lonza) supplemented with 10 μM ROCK inhibitor from day –13 to day –7. Growth medium was changed every 2–3 days until cells reached approximately 70%–90% confluence (day –7). Cells were harvested for electroporation 1 by trypsinization (EP1, described below). A total of 300,000 electroporated cells/well were seeded on HPC-coated 6-well tissue culture plates (Thermo Fisher Scientific) using seeding medium. After 24 hours, the seeding medium was replaced with complete BEGM supplemented with ROCK inhibitor (as described above), and cells were propagated from day –6 until cells reached approximately 70%–90% confluence (day 0). Cells were then harvested for electroporation 2 by trypsinization (EP2, described below). A total of 150,000–200,000 electroporated cells/well was seeded on HPC-coated Transwell inserts (Corning), with a diameter size of 6.5 mm and pore size of 0.4 μm, using seeding medium (added to apical and basolateral compartments). Twenty-four hours later, the apical medium was removed, and basolateral medium was replaced with ALI culture medium (described in supplemental methods). ROCK inhibitor was added until cells reached 100% confluence. Cells were grown at 37°C with 5% CO_2_, and medium was changed 3 times per week from day 0 until day 21. During the final week of ALI culture (day 21–28), the basolateral ALI medium of stimulated wells was supplemented with 10 ng/mL IL-13 (Peprotech). All analyses were performed on cells harvested on day 28.

### CRISPR/Cas9-targeting via electroporation.

CRISPR RNAs (crRNAs) targeting mature transcripts of hsa-miR-141-3p and the control gene SPDEF were designed using crispr.mit.edu. The UCSC Genome Browser (https://genome.ucsc.edu, hg38) CRISPR Targets 10K track was used to select crRNA sequences (efficiency score > 60% and MIT specificity > 50). crRNA sequences are shown in Supplemental Table 4, and hsa-miR-141 targeting is outlined in Supplemental Figure 1. The NT control crRNA was purchased readymade (Dharmacon). The crRNAs were prepared as previously described (21); details are provided in supplemental methods. In brief, we prepared the gRNA complex by combining crRNA (Dharmacon) with tracrRNA at a 1:1 ratio, allowing it to hybridize at 37°C for 30 minutes. The gRNA was then combined with recombinant Cas9 (MacroLab) and incubated at 37°C for 15 minutes yielding the ribonucleoprotein (RNP). An electroporation enhancer DNA oligonucleotide was added to the RNP to enhance efficiency of the delivery of the complex to the cells (48). The first round of electroporation (EP1) utilized the Neon transfection system (Invitrogen) (1,400 V, 2 pulses of 20 ms, protocol modified from ref. 49). A total of 300,000 cells was resuspended in 120 μL R buffer containing 5 μL RNP with the electroporation enhancer. The second round of electroporation (EP2) utilized the Amaxa Nucleofector 4D system (program DC-100; Lonza) where 150,000–200,000 cells were resuspended in 20 μL P3 Primary Cell Nucleofector Solution with Supplement 1 (P3 Primary Cell 4D NucleofectorTM X Kit S; Lonza) and mixed with 5 μL RNP supplemented with the electroporation enhancer.

### Analysis of CRISPR/Cas9 targeting efficiency.

Analysis of the targeting efficiency following CRISPR/Cas9 gene editing of ALI-cultured HBECs was performed as previously described (21). Briefly, genomic DNA was extracted using the RNA/DNA/Protein Purification Plus Kit (Norgen Biotek) according to the manufacturer’s protocol. Approximately 1 kb regions containing the gRNA targets were amplified using appropriate PCR primers (Supplemental Table 5) and Q5 DNA Polymerase (New England Biolabs), followed by purification using the QIAquick PCR Purification Kit (Qiagen). PCR products were analyzed employing Sanger sequencing (MCLAB) using sequencing primers listed in Supplemental Table 5. Sequencing files from cells treated with hsa-miR-141-3p-targeting gRNA, SPDEF-targeting gRNA, and NT gRNA controls were analyzed using the Interference of CRISPR Edits (ICE) tool by Synthego (50). Targeting efficiency was defined as percentage of indels (i.e., insertions or deletions).

### miRNA profiling by microarray and small RNA-seq.

Bronchial epithelial brushing samples from a controlled study of mild-to-moderate asthmatic subjects (*n* = 12–16) were sent for microarray as described earlier (16), and only the miR-141 data have been represented in this study (NCBI GEO database accession no. GSE34466). The samples (*n* = 16) were also submitted for miRNA-seq using a TruSeq platform (GSE164119). Abundance, assessed by total reads, of epithelial miRNAs run on a TruSeq platform was subsequently corrected using the following equation:

Corrected total reads = actual reads via TruSeq (average reads for all synthetic miRNAs on TruSeq/number of reads on TruSeq for a given synthetic miRNA).

The correction factor is derived from data of an equimolar synthetic miRNA pool run on TruSeq implementing an optimized protocol using a randomized 4 nucleotide sequence in the adaptor (NCBI GEO database accession number GSE94584) (51). Additionally, normalized reads of hsa-miR-141-3p from microarray of HBECs was used to study miR-141 expression levels over time in differentiating ALI cultures (*n* = 2 sample/time point; GSE164448).

### RNA extraction and qPCR.

Total RNA, including miRNA, was extracted from HBECs and mouse lung tissue (right middle lobe) using the RNA/DNA/Protein Purification Plus Kit (Norgen Biotek) according to the manufacturer’s protocol. RNA extraction from FAC-sorted HBEC populations (stored at –80°C in QIAzol) was carried out using the miRNeasy mini kit (Qiagen). RNA concentrations and quality were analyzed using the NanoDrop Spectrophotometer and 2100 Bioanalyzer (Agilent). For mRNA analysis, reverse transcription was performed using SuperScript VILO cDNA Synthesis Kit (Thermo Fisher Scientific). cDNA was preamplified using Advantage Polymerase (Takara Bio USA Inc.), and mRNA expression was normalized to housekeeping genes EEF1A1 (Eukaryotic Translation Elongation Factor 1 Alpha 1) and GAPDH. To enhance miRNA detection, reverse transcription was performed using miRNA-specific stem-loop primers and MultiScribe Reverse Transcriptase (Invitrogen). cDNA was preamplified using AmpliTaq Gold DNA Polymerase (Applied Biosystems). cDNA was purified using ExoSAP-IT (Applied Biosystems), followed by G-50 columns (GE Healthcare). miRNA expression was normalized to the expression of hsa-miR-103-3p and hsa-miR-191-5p (52). The quantitative PCR (qPCR) assay was performed on a ViiA 7 Real-Time PCR system (Thermo Fisher Scientific) using TaqMan Universal PCR Master Mix (Applied Biosystems). All primers and TaqMan probes are listed in Supplemental Table 5.

### RNA-seq and scRNA-seq.

RNA-seq libraries from 14 RNA samples of HBECs (*n* = 3–4/experimental condition), with high RNA integrity number (RIN) (range, 7.8–10), were generated using the NuGen Universal Plus mRNA kit (NuGen Technologies). The samples were sequenced using single-end 50 bp RNAseq on the HiSeq 4000 platform (GSE162696). GSEA was performed using GSEA software (53).

Allergic asthmatic subjects who underwent segmental allergen challenge for scRNA-seq were enrolled through the UCSF Airway Clinical Research Center (NCT02230189). For these analyses, we performed airway epithelial brushings at bronchoscopy in 4 participants with asthma 1 day after: (a) right middle lobe challenge with an allergen to which they were allergic (dust mite or cat) and (b) right upper lobe challenge with diluent, as a control, yielding *n* = 8 samples in all (GSE164015). We collected cells in PBS, centrifuged at 400g for 5 minutes at 4°C, resuspended them in Accumax solution (MilliporeSigma), disaggregated cells at room temperature using a rotating shaker, passed them through a 100 μM filter to ensure single cell dissociation, resuspend them in DMSO (54), cooled them gradually to –80°C in a cooling rack, and then transferred them to liquid nitrogen. All 8 samples were then thawed and processed in 1 batch using the 10× Chromium Next GEM Single Cell 3′GEM, Library & Gel Bead Kit and Chromium controller (10X Genomics). We recovered a mean of 4781 cells per sample and sequenced to a depth of approximately 300 million reads per sample. We performed initial preprocessing of these data, including singlet identification, alignment to the hg38 human genome, and UMI-based gene expression quantification, using Cell Ranger (Version 3.0, 10X Genomics). Overall, 91.2% of reads mapped to the genome. We performed subsequent analyses using Seurat v3.0 (55). We retained cells with > 200 and < 2500 features per cell and < 25% mitochondrial genes. After these quality control (QC) steps, we performed data normalization, feature selection (based on variability), scaling, sample integration, linear dimensional reduction, and clustering of cells using a shared nearest neighbor (SNN) modularity optimization-based clustering algorithm (*FindClusters* in Seurat) using a resolution parameter of 0.5, which yielded 18 cell clusters. Based on evaluation of cluster-specific cell markers, 7 of these clusters represented epithelial cell subpopulations and 11 represented immune cell populations. We annotated one of these clusters as goblet cells based on its complement of cluster-specific markers (Figure 5C).

### Flow cytometric analysis of epithelial subpopulations and MUC5AC.

HBECs grown on ALI filter inserts were harvested and fixed for flow cytometric analysis as described in supplemental methods. Antibody staining was performed by first blocking nonspecific binding using 2% FBS in PBS (FACS buffer) supplemented with 1:100 Trustain FcX (BioLegend) and 1:100 normal goat serum (Invitrogen) (blocking buffer) for 15 minutes at 4°C. Antibodies for surface staining (1:50–1:100 dilution, listed in Supplemental Table 3) were added to the cells in blocking buffer and incubated 30 minutes at 4°C, protected from light. Cells were washed in FACS buffer and permeabilized using 0.2% Saponin (Sigma-Aldrich) in blocking buffer for 30 minutes at 4°C. Antibodies for intracellular staining were added in the permeabilization buffer (1:100–1:200 dilution, listed in Supplemental Table 6) and incubated 1 hour at 4°C, protected from light. Cells were washed and resuspended in FACS buffer, followed by acquisition (20,000–100,000 events/sample) on a BD Biosciences LSRII Flow Cytometer running BD FACSDiva Software. Analysis was performed using FlowJo v10.6.1 (FlowJo LLC). In experiments involving FACS purification of nonpermeabilized live cells, cells were incubated with SiR-tubulin (Spirochrome, Cytoskeleton Inc.) according to the manufacturer’s recommendations before harvest, and cells were sorted using a BD FACSAria Cytometer the same day.

### Mouse model of Aspergillus-induced allergic asthma.

Age-matched female C57BL/6 mice at 3–4 months of age were used in the study. The mice were obtained from The Jackson Laboratory. Allergen-induced asthma was employed by intranasal administration of 100 μg *Aspergillus fumigatus* extract (Hollister-Stier Laboratories) in 40 μL sterile saline 3 times/week for 3 weeks (Figure 5A), described previously in ref. 56. Control, sham-challenged mice received sterile saline. On the third week, allergen-challenged mice were treated with 3 doses of mmu-miR-141-3p antagomir at 50 μg (days 14 and 16) and 100 μg (day 19) in 30 μL of sterile saline intranasally. The first dose of the antagomir was administered 6 hours prior to allergen challenge, and the remaining antagomir doses were administered 1 hour prior to allergen challenges. Scrambled antagomir was administered to a control group of allergen-challenged mice. Antagomir sequences are listed in Supplemental Table 2.

Airway hyperresponsiveness was assessed in response to increasing concentrations of acetylcholine using FlexiVent (Scireq) 48 and 72 hours after the last allergen challenge. BAL was collected for analysis of inflammatory infiltrates; lung tissue was processed for RNA extraction and PAS staining to assess goblet cell hyperplasia (described in supplemental methods).

### Statistics.

GraphPad Prism v8 was used for data analyses. For all figures, bar graphs and scatter dot plot data display mean ± SEM, unless otherwise stated. Before-and-after graphs demonstrate paired analyses, and 2-tailed paired *t* tests were used for statistical testing. For statistical tests of multiple groups, 1- or 2-way ANOVA was used with appropriate post hoc testing as stated in figure legends. A *P* value less than 0.05 was considered significant. All data were assumed to be distributed normally.

### Study approval.

The UCSF Committee on Human Research approved the use of primary airway epithelial cells isolated from human donor lungs, which were not used for lung transplantation. The cells were leftover clinical samples obtained from deidentified individuals and did not require written consent. UCSF IRB approval was obtained for microarray analysis (approval no. 10-03759) and qPCR and scRNA-seq analysis (approval no. 14-14224) of bronchial epithelial brushings. All animal experiments were approved by the UCSF IACUC (ethical permit no. AN182353).

## Author contributions

PGW, SS, and DJE contributed to study conception and design. SS, KJ, DJE, PGW, OLT, LRB, LZ, TLR, and KDK established original experimental methods. SS, KJ, OLT, LRB, LZ, AJ, SB, KDK, and RAB performed experiments. SS, KJ, AJ, SB, PGW, KMA, NRB, KDK, and OLT analyzed and interpreted data. PGW, DJE, WEF, AM, NRB, and KMA provided resources and supervised study execution. SS, KJ, and PGW drafted the original manuscript. All authors revised the manuscript. SS and KJ contributed equally to the study.

## Supplementary Material

Supplemental data

Supplemental Table 3

## Version 1 03/08/2021

Electronic publication
