# Supplementary material for: Epithelial miR-141 regulates IL-13–induced airway mucus production
Source: JCI Insight. 2021 Mar 8;6(5):e139019. doi: 10.1172/jci.insight.139019 (PMC8021117; doi:10.1172/jci.insight.139019)
Supplement: Supplemental data [file jciinsight-6-139019-s158.pdf]

Supplementary information

Epithelial miR-141 regulates IL-13-induced airway mucus production

Sana Siddiqui[*1,2], Kristina Johansson[*1,2,3], Alex Joo[1,2], Luke R. Bonser[4,5], Kyung Duk Koh[4,5], Olivier Le Tonqueze[4,5], Samaneh Bolourchi[1,2], Rodriel A. Bautista[1,2], Lorna Zlock[6], Theodore L. Roth[3,7,8,9], Alexander Marson[3,9,10,11,12,13], Nirav R. Bhakta[1], K. Mark Ansel[2,3], Walter E. Finkbeiner[6], David J. Erle[4,5], Prescott G. Woodruff[1,2,5,⁋]

[1]Department of Medicine, Division of Pulmonary and Critical Care Medicine, University of California, San Francisco, [2]Sandler Asthma Basic Research Center, University of California, San Francisco, [3]Department of Microbiology and Immunology, University of California, San Francisco, [4]Lung Biology Center, University of California, San Francisco, [5]Cardiovascular Research Institute, University of California, San Francisco, [6]Department of Pathology, University of California, San Francisco, [7]Biomedical Sciences Graduate Program, University of California, San Francisco, [8]Diabetes Center, University of California, San Francisco, [9]Innovative Genomics Institute, University of California, Berkeley, [10]J. David Gladstone Institutes, San Francisco,, [11]Department of Medicine, Division of Infectious Diseases, University of California, San Francisco, [12]Parker Institute for Cancer Immunotherapy, San Francisco and [13]Chan Zuckerberg Biohub, San Francisco, CA, USA

⁋Corresponding author
** Equal contribution*

Supplementary methods

Cell culture conditions

Culture plates and inserts were precoated with human placental collagen (15 μg/cm^2) (1). Human bronchial epithelial cells (HBECs) were seeded in medium that consisted of a 3:1 ratio of F12 and DMEM (MediaTech, ThermoFisher Scientific, Emeryville, CA) and was supplemented with 5% heat inactivated FBS, 100 U/ml penicillin, 100 μg/ml streptomycin, 10 μg/ml gentamicin, 250 ng/ml fungizone (UCSF Cell Culture Facility, San Francisco, CA), 5 μg/ml bovine insulin, 8.4 ng/ml cholera toxin, 25 ng/ml hydrocortisone (Sigma-Aldrich, St. Louis, MO) and 10 ng/ml rh-EGF (Atlanta Biologicals, Flowery Branch, GA). Rho-associated protein kinase inhibitor Y-27632 (10 μM, 'ROCK inhibitor', Enzo Life Sciences, Farmingdale, NY) was added right before use. Culture medium used for cells grown at air-

liquid-interface (ALI) consisted of 1:1 ratio of LHC Basal Medium and DMEM supplemented with 0.5 mg/ml BSA, 0.24 mg protein/ml BPE, 5 µg/ml bovine insulin, 10 µg/ml transferrin, 0.1 µM hydrocortisone (Sigma-Aldrich), 0.01 µM triiodothyronine, 2.7 µM epinephrine, 0.5 ng/ml rh-EGF (Atlanta Biologicals), 0.05 µM retinoic acid, 0.5 µM phosphorylethanolamine, 0.5 µM ethanolamine, 3 µM zinc sulfate, 100 U/ml penicillin, 100 µg/ml streptomycin and 2 mM L-glutamine (UCSF Cell Culture Facility).

Preparation of crRNAs

crRNAs were resuspended in 150 mM KCl and 10 mM Tris-HCl, pH 7.4. We prepared the ribonucleoprotein (RNP) complex by first incubating 160 µM, 1 µL of crRNA (Dharmacon, Lafayette, CO) with 160 µM, 1 µL tracrRNA, at 37°C for 30 min yielding 80 µM gRNA. The 80 µM gRNA was then added 1:1 with 40 µM, 2 µL rCas9, recombinant Cas9 (MacroLab, Berkeley, CA), yielding 20 µM RNP, which was incubated at 37°C for 15 min. An electroporation enhancer DNA oligonucleotide (100 µM, 1 µL) was added to the RNP to enhance efficiency of the delivery of the complex to the cells (2).

Preparation of HBECs for flow cytometry

ALI cultured HBECs were harvested on day 28 for analysis by flow cytometry. At harvest, 10 mM DTT (ThermoFisher Scientific) in PBS with $Ca^{2+}Mg^{2+}$ (Corning, Corning, NY) was added to the apical compartment and incubated for 10 min at 37°C. DTT wash was collected for dot blot analysis (described below). Cells were washes with PBS, then incubated for a maximum of 15 min at 37°C in 0.25% trypsin with 2.21 mM EDTA (Corning) which was added to the apical and basolateral compartments. Cell culture medium containing 5% FBS was added to neutralize the enzymatic activity and cells were washed in PBS followed by

fixation in 4% PFA (ThermoFisher Scientific) for 8 min on ice. Cells were washed in PBS and finally resuspended in plain PBS and stored at -80°C until analysis.

Secreted MUC5AC by dot blot

At harvest, the apical compartment of ALI cultured HBECs were washed with 10 mM DTT for 10 minutes at 37°C. The washes were stored at -80°C until analysis. Dot blot was adapted from the slot blot technique described earlier (3) and as performed previously (4). Following thawing, samples were diluted and spotted on to nitrocellulose. The membrane was allowed to dry, blocked in 4% milk, then stained with an anti-MUC5AC primary antibody (MAN-5ACI; gift from David J. Thornton, University of Manchester, Manchester, UK) (5). The blot was subsequently incubated with a HRP conjugated anti-rabbit secondary antibody and detected using TMB peroxidase substrate (Vector).

Histologic staining and immunofluorescence

6.5 mm ALI filters inserts were collected day 28 and placed in Carnoy's solution (6:3:1 ratios of methanol, chloroform, glacial acetic acid) for 30 min at RT as described previously (4). Briefly, filters were washed in concentrated methanol (2x20 min) followed by 4-5 washes in PBS. The filters were embedded in paraffin and cut in 5 μm sections that were later stained with Hematoxylin and Eosin (H&E), AB-PAS and fluorescent antibodies. H&E staining and AB-PAS staining was performed on deparaffinized sections according to standard protocol as previously described (6). Sections were hydrated and AB-PAS stained sections were placed in 3% acetic acid for 3 min followed by 1% alcian blue pH 2.5 for 30 min. Sections were then washed in tap water followed by DI water. 10 min incubation in 1% periodic acid was used to oxidize the sections, they were then washed in tap water and DI water. Sections were placed in Shiff's reagent for 20 min followed by 10 min wash in tap water and 30 sec incubation in

Mayer's Hematoxylin. 1 min wash in tap water was followed by 10 sec incubation in lithium carbonate. Sections were dehydrated according to standard protocol and mounted using Cytoseal^{TM} (ThermoFisher Scientific).

Immunofluorescence staining was done as previously described (6) using primary antibodies mouse monoclonal anti-MUC5AC (1:200 dilution) and rabbit polyclonal anti-MUC5B (1:200 dilution). After washing, slides where incubated with secondary antibodies Alexa Fluor® 488 goat anti-mouse and Alexa Fluor® 647 goat anti-rabbit (at 1:200 dilution for 2h, Jackson ImmunoResearch Laboratories, West Grove, PA) DAPI was used to stain nuclei.

Periodic-Acid Schiff (PAS) staining

Lung tissue from allergen-challenged mice treated with mmu-miR-141-3p antagomir or scrambled antagomir and saline-challenged control mice was fixed in formalin and processed as previously described (7). Assessment of PAS^{+} cells was performed on blinded lung tissue sections and analyzed using Image J Software (NIH, LOCI, University of Wisconsin). Airways with a basement perimeter (P_{BM}) >0.80 mm were considered as central airways ('large airways') and a P_{BM} <0.80 mm was considered peripheral airways ('small airways') and was based on a previous report (8).

Analysis of miR-141 gene targets

DIANA-microT (9,10) was used to obtain genomic coordinates of mmu-miR-141-3p binding sites of target genes predicted by TargetScan v.7.2 (8mer, 7merM8 and 7merA1 motifs). CLEAR-CLIP sequencing data was downloaded from the Gene Expression Omnibus (GEO) at GSE102716, published by Bjerke and Yi 2020 (11). Perfect overlap of genomic target sites and CLEAR-CLIP miR-141-3p peaks in wild type or miR-200 family induced epithelial cells,

and absence of miR-141-3p CLEAR-CLIP peak in miR-200 family deficient cells were considered experimentally confirmed miR-141 targets.

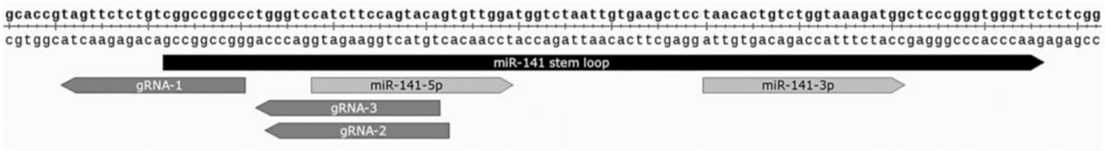

Supplementary Figure S1. CRISPR gRNA design for targeted knockdown of *MIR141* gene.

Human miR-141 stem loop is highlighted in black, mature hsa-miR-141-5p and hsa-miR-141-3p regions within the stem loop are highlighted in light grey and three CRISPR guide RNAs (gRNA-1, gRNA-2, gRNA-3) are shown in dark grey. Guide sequences are provided in supplementary Table S4.

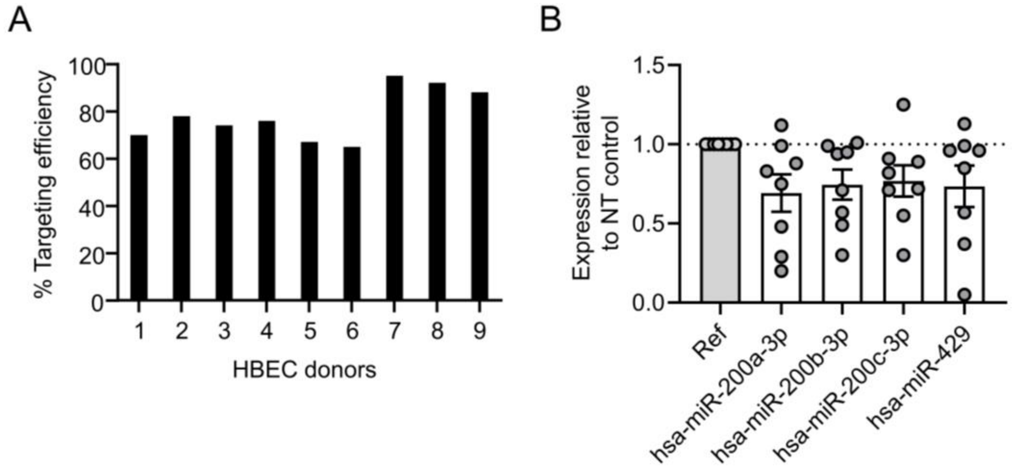

Supplementary Figure S2. Targeting efficiency and TaqMan qPCR.
A: Targeting efficiency of *MIR141* gRNAs assessed by Sanger DNA sequencing and ICE Synthego analysis (n=9 unique HBEC donors). **B:** Expression level of miR-141/200 family miRNAs (except hsa-miR-141, shown in Fig 2D) by TaqMan qPCR following administration of *MIR141*-targeting versus non-targeting (NT) gRNAs normalized to reference miRNAs hsa-miR-103a-3p and hsa-miR-191-5p ('Ref').

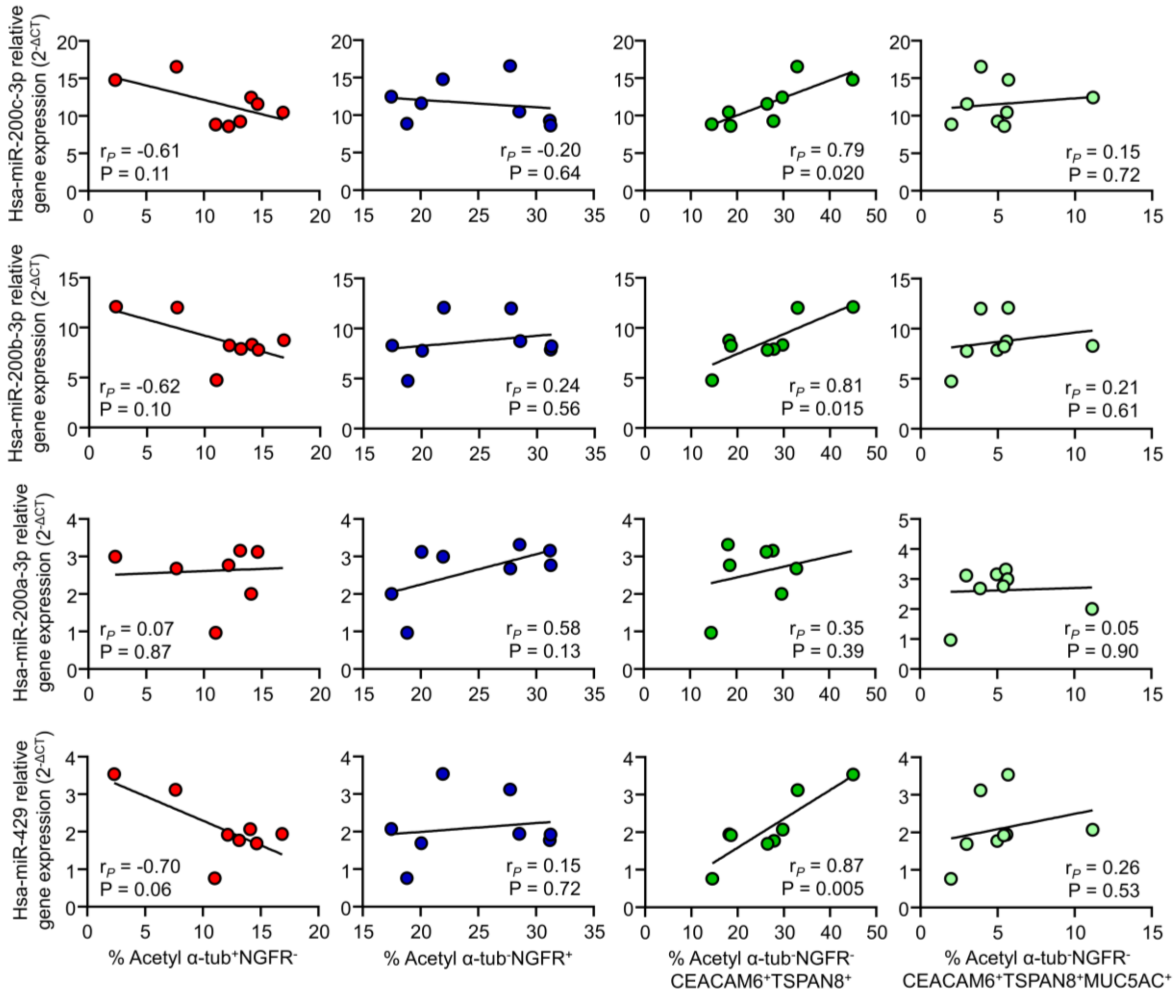

Supplementary Figure S3. Expression levels of miR-141/200 family miRNAs correlate with distinct epithelial subsets.

Frequency of ciliated cells (acetylated α-tubulin^{+}NGFR^{-}), basal cells (acetylated α-tubulin^{-}NGFR^{+}), secretory cells (acetylated α-tubulin^{-}NGFR^{-}CEACAM6^{+}TSPAN8^{+}) and mucus-producing goblet cells (acetylated α-tubulin^{-}NGFR^{-}CEACAM6^{+}TSPAN8^{+}MUC5AC^{+}) assessed by Flow cytometry are plotted against the expression level of miR-141/200 family miRNAs determined by TaqMan qPCR. Epithelial cells were grown at air-liquid interface (ALI) with IL-13. N=8. r_P, Pearson correlation coefficient.

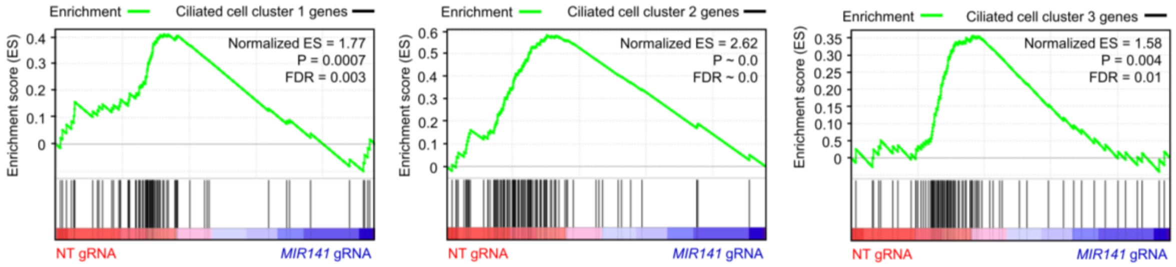

Supplementary Figure S4. Gene set enrichment analysis of ciliated cell gene clusters. Single cell RNA sequencing analysis of bronchial epithelial brushings obtained from allergic asthmatic subjects identified distinct epithelial cell clusters. Gene set enrichment analysis of positively expressed ciliated cell genes in cluster 1, 2 and 3 (provided in Supplementary Table S3) in HBEC cultures that have undergone gene-editing with non-targeting (NT) or *MIR141* gRNA, subsequently grown at air-liquid-interface (ALI) with IL-13 stimulation.

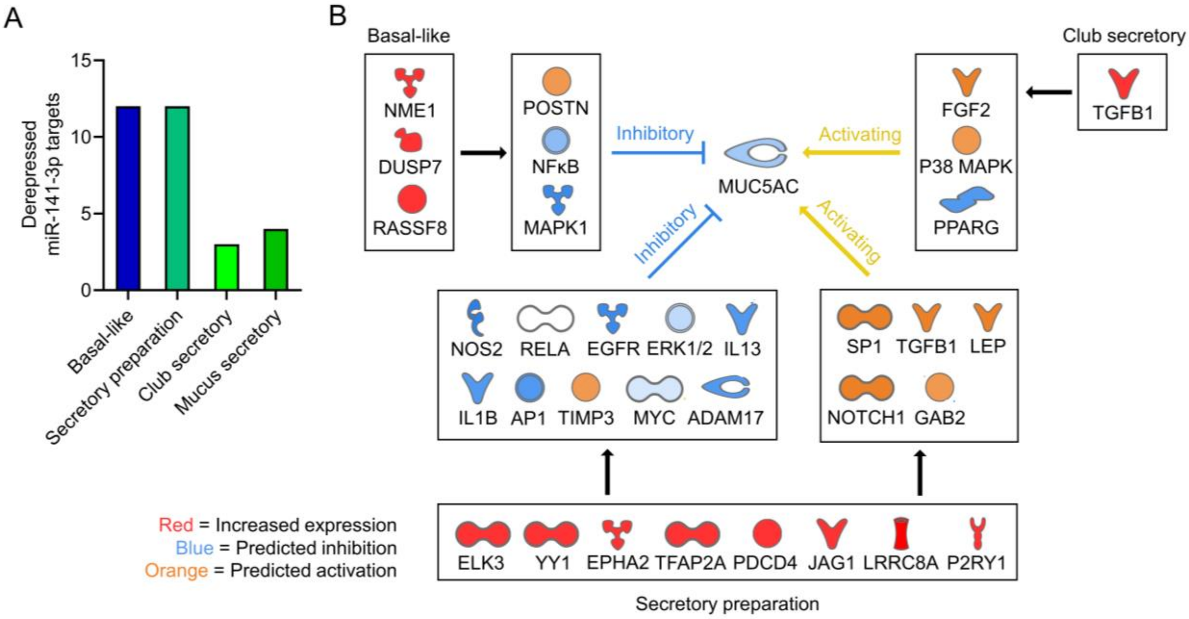

Supplementary Figure S5. Net effect of miR-141-target gene derepression by IPA predicts inhibition of MUC5AC expression.
A: Most derepressed miR-141-3p-target genes in HBECs that have undergone gene-editing with *MIR141* gRNAs compared to NT controls belongs to early basal-like and secretory preparation clusters (determined by pseudotime gene expression analysis (12)). **B:** Ingenuity Pathway Analysis (IPA) of connecting derepressed genes (increased activity, red) to downstream MUC5AC expression identifies inhibitory (light blue) and activating (yellow) pathways. Net effect of increased activity of derepressed target genes results in inhibition of MUC5AC expression (light blue).

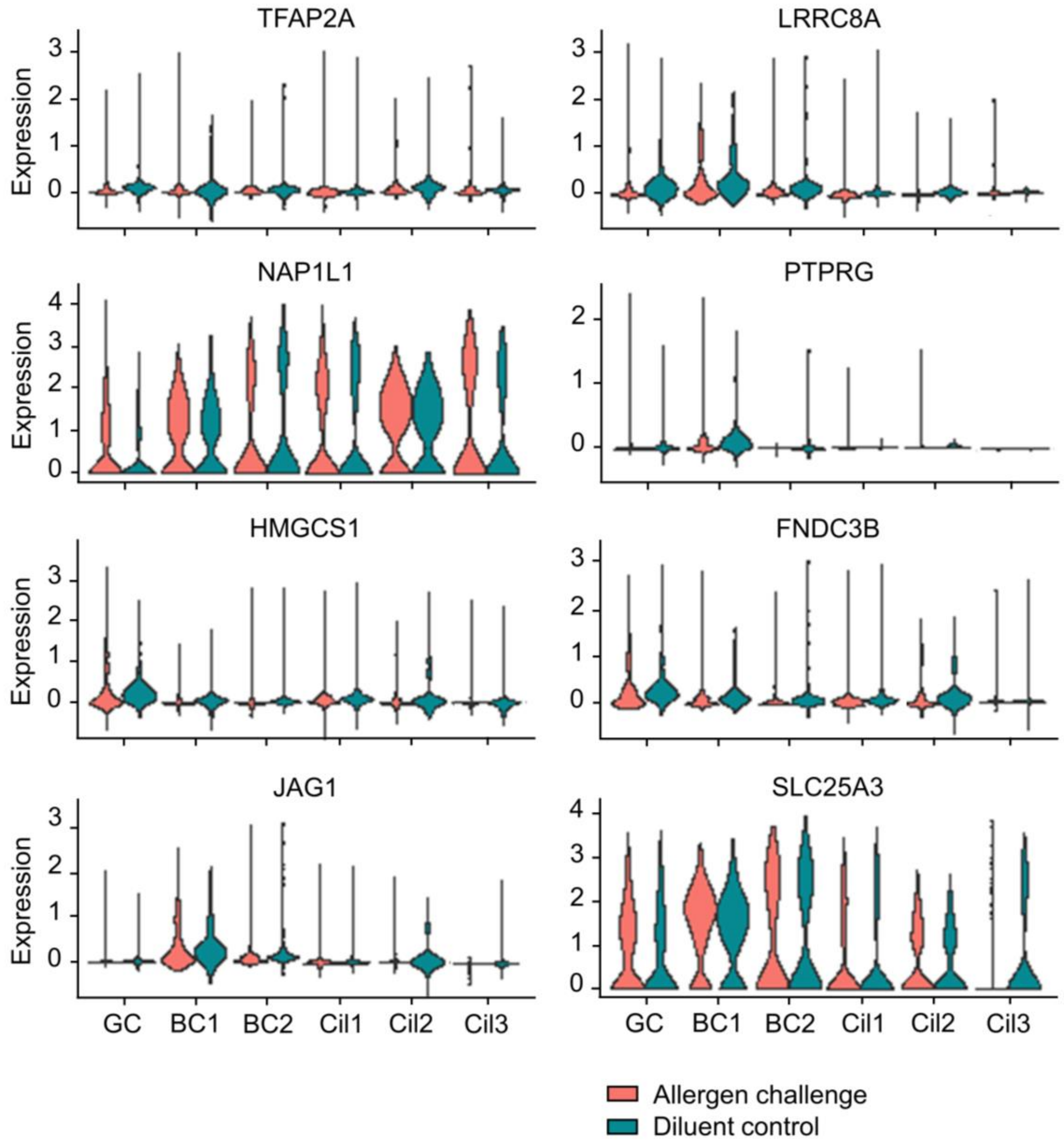

Supplementary Figure S6. miR-141 target genes broadly detected in epithelial subpopulations by scRNA-seq.

Gene expression by single cell RNA sequencing of bronchial epithelial brushings obtained from allergic asthmatic subjects 24h post segmental allergen challenge (n=4) or diluent control (n=4).

Table S1. Patient demographics TaqMan qPCR (Figure 1C-E).

**Subject**	**1**	**2**	**3**	**4**	**5**	**6**	**7**
**Age**	29	24	30	42	18	34	29
**Sex**	F	F	M	F	M	M	F
**Race**	White	Asian	White	White	White	White	White
**BMI (kg/m^2)**	25.7	32.0	24.5	25.6	26.6	26.4	38.4
**Allergen**	Cat	HDM	HDM	HDM	HDM	HDM	HDM
**Serum IgE (IU/mL)**	102	226	30	30	343	149	68
**FEV1 (L)**	4.10	2.90	3.76	2.64	4.02	4.25	3.21
**FEV1 (%)**	116.6	100.2	79.7	91.5	110.8	93.5	94.4
**FVC (L)**	4.62	3.85	5.39	3.49	5.48	6.57	3.82
**FVC (%)**	110.4	115.6	95.1	98.4	132.4	116.3	94.5
**FeNO (ppb)**	33	24	39	32	42	19	18
**Blood Eosinophils (10^9/L)**	0.07	0.16	0.13	0.08	0.06	0.12	0.16

Forced expiratory volume in one second, FEV1; Forced vital capacity, FVC; House dust mite, HDM.

Table S2. Patient demographics scRNA-seq (Figure 5A-C; Figure 6; Figure 7F; Supplementary Figure S6; Supplementary Table S3).

**Subject**	**1**	**2**	**3**	**4**
**Age**	42	29	24	18
**Sex**	M	F	M	M
**Race**	White	Declined to specify	Black or African American	White
**BMI (kg/m^2)**	18.9	24.8	24.8	26.6
**Allergen**	HDM	Cat	HDM	HDM
**Serum IgE (IU/mL)**	85	433	695	343
**FEV1 (L)**	3.33	2.91	3.98	4.02
**FEV1 (%)**	77.2	95.6	112.5	110.8
**FVC (L)**	5.56	3.74	4.62	5.48
**FVC (%)**	101.8	104.8	111.4	132.4
**FeNO (ppb)**	77	9	67	42
**Blood Eosinophils (10^9/L)**	0.19	0.06	0.24	0.06

Forced expiratory volume in one second, FEV1; Forced vital capacity, FVC; House dust mite, HDM.

Table S3. scRNA-seq clusters (Figure 5A-C; Figure 6; Figure 7F; Supplementary Figure S6).

Provided as Excel spreadsheet.

Table S4. crRNA sequences.

Name	crRNA target DNA sequence (5'-3')	Manufacturer
Hsa-miR-141-3p gRNA-1	GGCCGGCCGACAGAGAACTA	Dharmacon
Hsa-miR-141-3p gRNA-2	CTGTACTGGAAGATGGACCC	Dharmacon
Hsa-miR-141-3p gRNA-3	TGTACTGGAAGATGGACCCA	Dharmacon
SPDEF-1	GGAGAGCTGGACCGACAGCG	Dharmacon
SPDEF-2	ATGAAGCGGCCATAGCTGTG	Dharmacon
Non-targeting control (NT)	GATACGTCGGTACCGGACCG	Dharmacon CatalogU-007501-01-05

Table S5. Sequences for PCR primers, DNA sequencing primers, RT-PCR primers and antagomirs used in the study.

Name	Primer sequence (5'-3')	Manufacturer	Application
miR-141-Forward	TCGTCTTACCCAGCAGTGTTTG	Integrated DNA Technologies	Amplification of target region
miR-141-Reverse	ACCTGAATCTCCCACTACTGC	Integrated DNA Technologies	Amplification of target region
SPDEF-Forward	CTCACTTGGCAAGAGCATCC	Integrated DNA Technologies	Amplification of target region
SPDEF-Reverse	CCATGTCAGATGTCCTCATCTG	Integrated DNA Technologies	Amplification of target region
miR-141-seq	TCTTGAGCTGAGAGCGTTGC	Integrated DNA Technologies	Sequencing, editing efficiency
SPDEF-seq	TGTCCCATGAGAGCTGCATA	Integrated DNA Technologies	Sequencing, editing efficiency
hsa/mmu-miR-141-3p-Forward	ACACTCCAGCTGGGTAACACTGTCTGGTAA	Integrated DNA Technologies	miRNA expression
hsa/mmu-miR-141-3p-Reverse	CTCAACTGGTGTCGTGGAGTCGGCAATTCAGTTGAGCCATCTTT	Integrated DNA Technologies	miRNA expression
hsa/mmu-miR-141-3p-Probe	TTCAGTTGAGCCATCTTT	Integrated DNA Technologies	miRNA expression
hsa-miR-200a-3p-Forward	ACACTCCAGCTGGGTAACACTGTCTGGTAA	Integrated DNA Technologies	miRNA expression
hsa-miR-200a-3p-Reverse	CTCAACTGGTGTCGTGGAGTCGGCAATTCAGTTGAGACATCGTT	Integrated DNA Technologies	miRNA expression
hsa-miR-200a-3p-Probe	TTCAGTTGAGACATCGTT	Integrated DNA Technologies	miRNA expression
hsa-miR-200b-3p-Forward	ACACTCCAGCTGGGTAATACTGCCTGGTAA	Integrated DNA Technologies	miRNA expression
hsa-miR-200b-3p-Reverse	CTCAACTGGTGTCGTGGAGTCGGCAATTCAGTTGAGTCATCATT	Integrated DNA Technologies	miRNA expression
hsa-miR-200b-3p-Probe	TTCAGTTGAGTCATCATT	Integrated DNA Technologies	miRNA expression
hsa-miR-200c-3p-Forward	ACACTCCAGCTGGGTAATACTGCCGGGTAAT	Integrated DNA Technologies	miRNA expression
hsa-miR-200c-3p-Reverse	CTCAACTGGTGTCGTGGAGTCGGCAATTCAGTTGAGTCCATCAT	Integrated DNA Technologies	miRNA expression
hsa-miR-200bc-3p-Probe	TTCAGTTGAGTCCATCAT	Integrated DNA Technologies	miRNA expression
hsa-miR-429-Forward	ACACTCCAGCTGGGTAATACTGTCTGGTAA	Integrated DNA Technologies	miRNA expression
hsa-miR-429-Reverse	CTCAACTGGTGTCGTGGAGTCGGCAATTCAGTTGAGACGGTTTT	Integrated DNA Technologies	miRNA expression
hsa-miR-429-Probe	TTCAGTTGAGACGGTTTT	Integrated DNA Technologies	miRNA expression
mmu-miR-429-Forward	ACACTCCAGCTGGGTAATACTGTCTGGTAA	Integrated DNA Technologies	miRNA expression
mmu-miR-429-Reverse	CTCAACTGGTGTCGTGGAGTCGGCAATTCAGTTGAGACGGCATT	Integrated DNA Technologies	miRNA expression
mmu-miR-429-Probe	TTCAGTTGAGACGGCATT	Integrated DNA Technologies	miRNA expression
hsa-miR-103a-3p/mmu-miR-103-3p Forward	ACACTCCAGCTGGGAGCAGCATTGTACAGGG	Integrated DNA Technologies	miRNA expression
hsa-miR-103a-3p/mmu-miR-103-3p Reverse	CTCAACTGGTGTCGTGGAGTCGGCAATTCAGTTGAGTCATAGCC	Integrated DNA Technologies	miRNA expression

hsa-miR-103a-3p/mmu-miR-103-3p Probe	TTCAGTTGAGTCATAGCC	Integrated DNA Technologies	miRNA expression
hsa/mmu-miR-191-5p Forward	ACACTCCAGCTGGGCAACGGAATCCCAAAAG	Integrated DNA Technologies	miRNA expression
hsa/mmu-miR-191-5p Reverse	CTCAACTGGTGTCGTGGAGTCGGCAATTCAGTTGAGCAGCTGCT	Integrated DNA Technologies	miRNA expression
hsa/mmu-miR-191-5p Probe	TTCAGTTGAGCAGCTGCT	Integrated DNA Technologies	miRNA expression
Universal Reverse	CTCAAGTGTCGTGGAGTCGGCA	Integrated DNA Technologies	miRNA expression
m-MUC5AC-Forward	GTCCAAGGAAAGTGAGGAACATG	Integrated DNA Technologies	Mouse mRNA expression
m-MUC5AC-Reverse	TACTGGAAAGGCCCAAGCAT	Integrated DNA Technologies	Mouse mRNA expression
m-MUC5AC-Probe	CCTCGCTGACCCTGAATGCCAACT	Integrated DNA Technologies	Mouse mRNA expression
m-Clca3- Forward	CTTCGGATCAGGTTCAGAACAAT	Integrated DNA Technologies	Mouse mRNA expression
m-Clca3- Reverse	CGATCGCCGCATTTCC	Integrated DNA Technologies	Mouse mRNA expression
m-Clca3-Probe	TGTTGATGCTTTCGCAGCACTCTCCTC	Integrated DNA Technologies	Mouse mRNA expression
Mmu-anti-miR-141-3p Antagomir	mC*mC*mAmUmCmUmUmUmAmCmCmAmGmAmCmAmGmUmG*mU*mU*mA*-Chl	Dharmacon	In vivo inhibition
Scrambled Antagomir	mU*mC*mAmCmAmAmCmCmUmCmCmUmAmGmAmAmAmGmA*mG*mU*mA*3'-Chl	Dharmacon	In vivo inhibition control

Antagomir sequence: mN, 2′OMe base; *, phosphorothioate linkage; Chl, cholesterol

Table S6. Antibodies.

Antigen	Clone	Fluorochrome	Manufacturer	Application
Acetylated α tubulin	6-11 B-1	Alexa Fluor® 488	Santa Cruz Biotechnology	Flow cytometry
MUC5AC	45M1	DyLight 488, PE	Novus Biologicals	Flow cytometry
CD66c/CEACAM6	B6.2/CD66	BV786	BD Biosciences	Flow cytometry
CD271/NGFR	ME20.4	PE-Cy7	BioLegend	Flow cytometry
TSPAN8	FAB4734N	Alexa Fluor® 700	R&D Systems	Flow cytometry
MUC5AC	MAN-5ACI	n/a	Gift from Thornton lab, University of Manchester, Manchester, UK	Dot blot
MUC5AC	45M1	n/a	ThermoFisher Scientific	Immunofluorescence
MUC5B	H-300	n/a	Santa Cruz Biotechnology	Immunofluorescence
Goat-anti rabbit	n/a	Alexa Fluor® 647	Jackson ImmunoResearch Laboratories	Immunofluorescence
Goat anti-mouse	n/a	Alexa Fluor® 488	Jackson ImmunoResearch Laboratories	Immunofluorescence

Supplementary references

1. Coleman DL, Tuet IK, Widdicombe JH. Electrical properties of dog tracheal epithelial cells grown in monolayer culture. *Am J Physiol*. 1984;246(3 Pt 1):C355-359.

2. Roth TL, et al. Reprogramming human T cell function and specificity with non-viral genome targeting. *Nature*. 2018;559(7714):405–9.

3. Harrop CA, Thornton DJ, McGuckin MA. Detecting, Visualising, and Quantifying Mucins. *Methods Mol Biol*. 2012;842:49-66.

4. Bonser LR, Zlock L, Finkbeiner W, Erle DJ. Epithelial tethering of MUC5AC-rich mucus impairs mucociliary transport in asthma. *J Clin Invest.* 2016;126(6):2367–71.

5. Sheehan JK, et al. Physical characterization of the MUC5AC mucin: a highly oligomeric glycoprotein whether isolated from cell culture or in vivo from respiratory mucous secretions. *Biochem J*. 2000;347(Pt 1):37–44.

5. Koh KD, et al. Efficient RNP-Directed Human Gene Targeting Reveals SPDEF is Required for IL-13-Induced Mucostasis. *Am J Respir Cell Mol Biol*. 2020;62(3):373-381.

7. Siddiqui S, et al. The modulation of large airway smooth muscle phenotype and effects of epidermal growth factor receptor inhibition in the repeatedly allergen-challenged rat. *Am J Physiol Lung Cell Mol Physiol*. 2013;304(12):L853–62.

8. Pinelli V, Marchica CL, Ludwig MS. Allergen-induced asthma in C57Bl/6 mice: hyper-responsiveness, inflammation and remodelling. *Respir Physiol Neurobiol*. 2009;169(1):36–43.

9. Reczko M, Maragkakis M, Alexiou P, Grosse I, Hatzigeorgiou AG. Functional microRNA targets in protein coding sequences. *Bioinformatics*. 2012;28(6):771–6.

10. Paraskevopoulou MD, Georgakilas G, Kostoulas N, Vlachos IS, Vergoulis T, Reczko M, et al. DIANA-microT web server v5.0: service integration into miRNA functional analysis workflows. *Nucleic Acids Res*. 2013;41(Web Server issue):W169-173.

11. Bjerke GA, Yi R. Integrated analysis of directly captured microRNA targets reveals the impact of microRNAs on mammalian transcriptome. *RNA*. 2020;26(3):306–23.

12. Goldfarbmuren KC, et al. Dissecting the cellular specificity of smoking effects and reconstructing lineages in the human airway epithelium. *Nat Commun*. 2020;11(1):2485.
